# Synthesis of Cu_2_Se-Based Materials and Their Application in Energy Conversion and Storage

**DOI:** 10.3390/molecules30204074

**Published:** 2025-10-13

**Authors:** Kai Zhang, Songjun Li, Maiyong Zhu

**Affiliations:** School of Materials Science & Engineering, Jiangsu University, Zhenjiang 212013, China

**Keywords:** thermoelectric, energy storage, Cu_2_Se

## Abstract

With environmental pollution and energy shortages becoming increasingly severe, developing efficient energy conversion and storage technologies is crucial. Cu_2_Se has garnered significant attention as a thermoelectric material due to its abundant raw materials, low cost, and high thermoelectric figure of merit (ZT). This paper reviews the synthesis methods and application progress of Cu_2_Se in the energy field. Regarding synthesis, various methods such as solid-state synthesis, hydrothermal synthesis, and ion exchange can be employed to control its microstructure and properties. In applications, Cu_2_Se demonstrates significant potential in thermoelectric conversion by harnessing the Seebeck effect to convert waste heat into electricity. Simultaneously, its high carrier mobility and favorable electrochemical properties make it promising for energy storage systems like sodium-ion batteries and aqueous batteries. Furthermore, this material holds considerable potential in emerging fields such as flexible wearable devices and high-efficiency thermoelectric power generation systems. Future research should continue optimizing its comprehensive properties to advance the practical application of Cu_2_Se in energy conversion and storage.

## 1. Introduction

**Figure 1:**
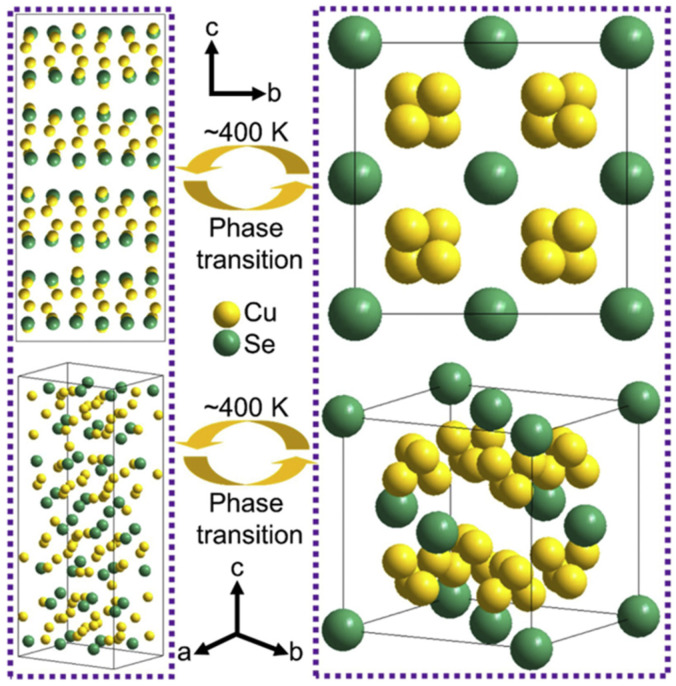
Schematic crystal structures of low-temperature monoclinic Cu_2_Se and high-temperature antifluorite Cu_2_Se. Reproduced from ref. [7] with permission. Copyright 2020 Elsevier.

As global environmental pollution and energy shortages intensify, developing efficient and sustainable energy technologies has become a critical challenge facing humanity. The non-renewable nature of traditional fossil fuels and the greenhouse gases emitted during their use have profoundly impacted ecosystems. While clean energy sources like wind and solar power hold immense potential, their low energy density and intermittent nature limit their ability to meet continuously growing energy demands [1]. Against this backdrop, thermoelectric technology has garnered significant attention for its ability to directly convert waste heat into electricity. Among these, Cu_2_Se, as an emerging thermoelectric material, has emerged as an ideal alternative to traditional lead-containing materials (e.g., PbTe) due to its unique physicochemical properties and environmental friendliness. In recent years, novel thermoelectric materials like poly (3-hexylthiophene)-based materials [2], MXene-based materials [3] and graphene-based materials [4] have demonstrated broad application prospects. Research on optimizing the thermoelectric properties, interface engineering, and composite structure design of these materials provides important references for understanding the multifunctional characteristics of Cu_2_Se. Compared with traditional thermoelectric materials (e.g., Bi_2_Te_3_, PbTe), Cu_2_Se exhibits significant advantages. The structural properties of its low-temperature monoclinic phase (α-Cu_2_Se) and high-temperature antifluorite phase (β-Cu_2_Se) endow it with extremely low lattice thermal conductivity and ultra-high electrical conductivity, and the crystal structures of the low-temperature monoclinic Cu_2_Se and the high-temperature antifluorite-phase Cu_2_Se are schematically shown in Figure 1. In β-Cu_2_Se, the liquid-like migration behavior of Cu^+^ ions effectively enhances the phonon scattering, which enables the material to maintain excellent thermoelectric properties at high temperatures (up to a ZT value of 2.13 [5]), while the raw material cost is much lower than that of Bi_2_Te_3_ [6]. In addition, the narrow bandgap (~1.2 eV) and high Cu^+^ activity of Cu_2_Se enable it to perform equally well in the field of energy storage, e.g., the nitrogen-doped carbon-covered Cu_2_Se@NC composites retained a high capacity of 246.8 mAh g^−1^ in sodium-ion batteries after 2500 cycles at a current density of 10 A g^−1^.

The dual function of Cu_2_Se in the energy field is reflected in its integrated potential of “waste heat to power–power storage”. Through the Seebeck effect, Cu_2_Se can convert industrial waste heat into electricity; at the same time, it can be used as an electrode material for sodium-ion batteries or aqueous batteries to realize energy storage. For example, Cu_2_Se nanosheets (E-Cu_2_Se) were used as the anode in aqueous zinc-ion batteries, and ZnxMnO_2_ as the cathode in flexible aqueous zinc-ion batteries, which maintained a capacity of 40 mAh g^−1^ after 60,000 cycles [8]. This synergistic effect provides a new idea for constructing self-powered energy systems, especially in wearable electronic devices, and Cu_2_Se-based thermoelectric-energy storage integrated devices show a broad application prospect [9].

**Figure 2:**
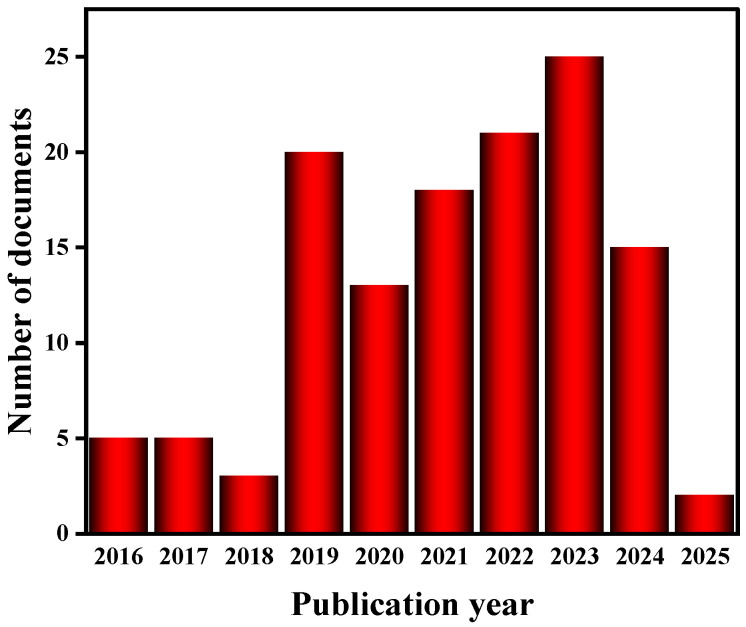
Application of copper–selenium-based materials in batteries in the last decade.

Although copper sulfide-based materials (e.g., CuS, Cu_2_S) have been extensively studied in the field of thermoelectricity, a systematic review of copper selenide-based materials (Cu_2_Se) in energy conversion and storage is still lacking. The existing literature mostly focuses on a single application scenario, e.g., Zhang et al. [10] systematically summarized the interfacial engineering strategy of copper sulfide nanomaterials in thermoelectric power generation, while Yang et al. [11] pioneered the proposal of Cu-based ternary heterostructures for asymmetric supercapacitors; their research system is still dominated by sulfides. Selenides (especially Cu_2_Se) are less reported, especially in the literature on supercapacitors (Web of Science statistics show that there are only 15 relevant papers), and the application of Cu_2_Se-based materials in energy storage is mainly focused on batteries, especially sodium-ion batteries and aqueous batteries. This manuscript provides a comprehensive and detailed analysis of Cu_2_Se’s applications in thermoelectric materials. It also compiles a decade’s worth of literature on Cu_2_Se-based batteries (Figure 2), categorizing them into sodium-ion batteries and aqueous batteries. The current situation highlights two core issues in the field: (1) the multifunctional properties of Cu_2_Se-based materials have not been fully explored, and the synergistic mechanism across energy conversion and storage has not been systematically analyzed; (2) the existing studies are mostly limited to a single performance optimization, and there is a lack of a whole-chain design perspective from the synthesis of materials to the integration of devices.

This manuscript aims to fill this gap by comprehensively analyzing copper selenide-based materials from synthesis to application, with a focus on their dual functionality in thermoelectric conversion and electrochemical energy storage. We first review primary synthesis methods, including solid-state, hydrothermal/solvothermal, ion exchange, template-directed, and chemical reduction approaches. Subsequently, we explore pathways to enhance the thermoelectric properties of Cu_2_Se through a series of strategies. We explore the integration potential of copper selenides in emerging applications such as flexible wearables and self-powered systems, while also highlighting Cu_2_Se’s role in energy storage. Through interdisciplinary convergence and technological innovation, Cu_2_Se holds promise as a core material for next-generation energy systems, accelerating humanity’s progress toward a sustainable future characterized by zero waste heat and zero emissions.

## 2. Synthetic Strategies

**Figure 3:**
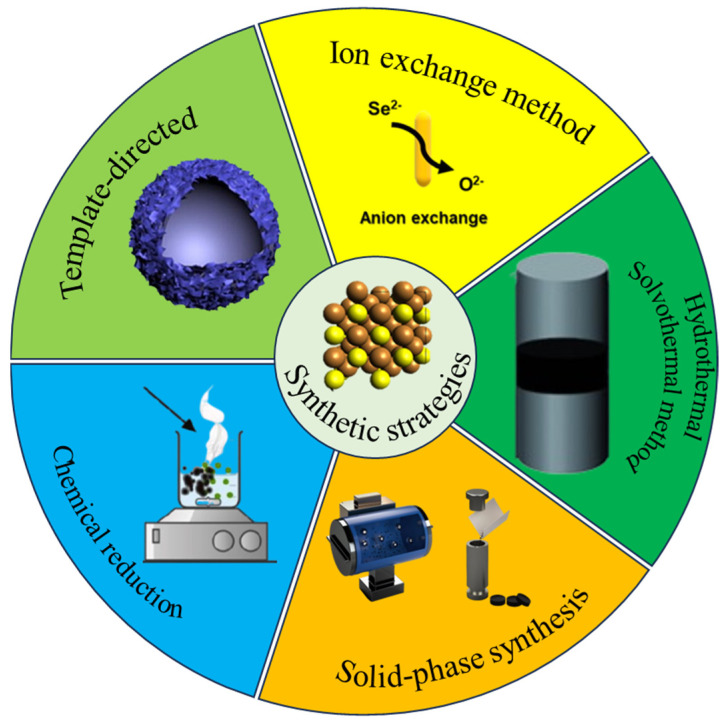
Summary diagram of synthesis methods.

Cu_2_Se has been synthesized by various methods, such as pulsed laser deposition, solid-phase synthesis, hydrothermal/solvent-thermal, ion exchange, template-directed, chemical reduction, vacuum evaporation, electrochemical deposition [12], and magnetron sputtering. Figure 3 introduces the main synthesis methods of Cu_2_Se.

### 2.1. Template-Directed

The template-directed method is often used for the preparation of hollow-structured nanomaterials, especially in the preparation of hollow spheres. The template-directed method is accompanied by ion exchange reaction, diffusion, coordinated etching and precipitation (CEP), etc. Generally, a specific nanostructured material is selected as a template first, and then the material is deposited on the surface of the template through a series of means, and the final product obtained is very similar to the original template in terms of shape and size. The advantage of the template-directed method is that the size and shape of the product can be precisely controlled by the precise selection of the size and shape of the original template, so as to achieve precise control of the product. In addition, as a common synthesis method, the template-directed method is often used to synthesize heterojunction materials because the synthesized new materials can retain the size and shape of the original materials well. In short, a heterojunction exists as a special type of PN junction within semiconductors, typically formed by sequentially depositing two layers of different semiconductor materials on the same substrate. Due to the differing bandgaps of these materials, the presence of both positive charge carriers (holes) and negative charge carriers (electrons) increases the recombination rate within the diode. This effect brings the ideal diode very close to the characteristics of a real diode.

Of course, more than two semiconductor materials can also be made heterojunction, but it is relatively rare. Heterojunctions can be used to make light-emitting components, diodes, transistors, and so on.

**Figure 4:**
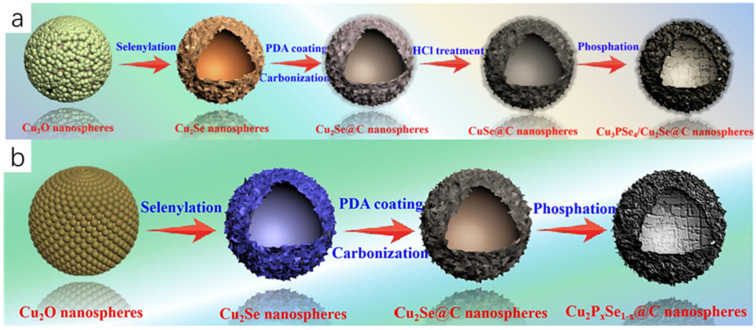
(**a**) Flow chart for the preparation of Cu_3_PSe_4_/Cu_2_Se@C nanospheres. Reproduced from ref. [13] with permission. Copyright 2023 Elsevier. (**b**) Flow chart for the preparation of Cu_2_P_x_Se_1−x_@C nanorods. Reproduced from ref. [14] with permission. Copyright 2023 Elsevier.

Zhao et al. [13] finally obtained carbon-encapsulated 3D hollow heterostructured Cu_3_PSe_4_/Cu_2_Se nanospheres (Cu_3_PSe_4_/Cu_2_Se@C) by a template-directed method using morphologically regular Cu_2_O nanospheres as templates and precursors through a series of steps: selenization, carbon-encapsulation, and phosphorylation. Firstly, Cu_2_O nanospheres were prepared by hydrothermal method using Cu(CH_3_COO)_2_·H_2_O, PVP, glucose, and dimethylformamide (DMF) as raw materials, and then selenide was performed on the Cu_2_O nanospheres to obtain the black-precipitated hollow Cu_2_Se nanospheres, and then dopamine hydrochloride was reacted with the hollow Cu_2_Se nanospheres to obtain the hollow Cu_2_Se@C nanospheres, and the hollow Cu_2_Se@C nanorods were immersed in hydrochloric acid solution to obtain pure CuSe@C hollow nanorods, and finally phosphorylated with sodium hypophosphite NaH_2_PO_2_ to obtain the final product of hollow Cu_3_PSe_4_/Cu_2_Se@C nanorods (Figure 4a). On the same principle, Zhao et al. [14] prepared hollow Cu_2_P_x_Se_1−x_@C nanorods through a series of steps including selenization, carbonization, and phosphorylation (Figure 4b).

Yang et al. [15] successfully prepared Ni(OH)_2_@Cu_2_Se hollow heterostructures by reacting Ni(OH)_2_ with Cu_2_Se nanosheets using a template-oriented method. The working group used Cu_2_O as a template, added Cu_2_O and NiCl_2_ into a mixed solution of water and alcohol, sonicated to ensure a homogeneous mixture, and added PVP and Na_2_S_2_O_3_; a large number of Ni^2+^ particles were absorbed by Cu_2_O under the synergistic effect of PVP, and the reaction of Cu_2_O with S_2_O_3_^2−^ led to the OH^−^ and [Cu_2_(S_2_O_3_^2−^)_x_]^2−2x^ ion. A large number of OH^−^ and [Cu_2_(S_2_O_3_^2−^)_x_]^2−2x^ ions appeared in large quantities, and cooperative etching and precipitation (CEP) appeared; Ni(OH)_2_ diffused into the outer layer to encapsulate the Cu_2_O, and Cu_2_O@Ni(OH)_2_ was formed. Subsequently, the solution obtained by mixing Se powder and NaBH4 was dropped into the Cu_2_O@Ni(OH)_2_ solution, and then Na_2_S_2_O_3_ solution was added. Under the action of CEP, [Cu_2_(S_2_O_3_^2−^)_x_]^2−2x^ ions diffused to the outer layer and interacted with the HSe-ions to produce Cu_2_Se wrapped around the outer layer of Cu_2_O@Ni(OH)_2_, and Ni(OH)_2_@Ni(OH)_2_@Cu_2_Se was formed; finally, Na_2_S_2_O_3_ reacted with Cu_2_O, forming the final product, which was the Ni(OH)_2_@Cu_2_Se hollow heterostructure.

The chemical reaction equations involved in the reaction are as follows:

Cu_2_O + XS_2_O_3_^2−^ + H_2_O → [Cu_2_(S_2_O_3_^2−^)_x_]^2−2x^ + 2OH^−^ (1)

Ni^2+^ + 2OH^−^ → Ni(OH)_2_ (2)

[Cu_2_(S_2_O_3_^2−^)_x_]^2−2x^ + HSe^−^ + (x − 1)H_2_O → Cu_2_Se + xHS_2_O_3_^−^ + (x − 1)OH^−^ (3)

**Figure 5:**
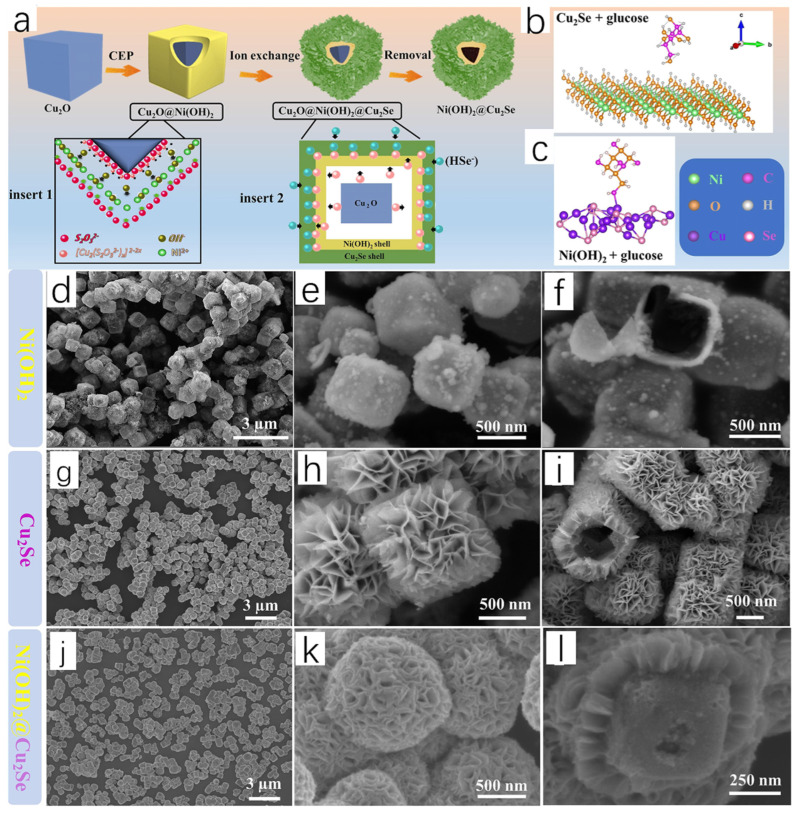
(**a**) Schematic diagram of Ni(OH)_2_@Cu_2_Se HHSs prepared according to the ion exchange principle. (**b**) Glucose adsorbed Ni(OH)_2_ structure. (**c**) Glucose adsorbed Cu_2_Se structure. (**d**–**f**) High-power transmission electron microscope images of Ni(OH)_2_ NCs. (**g**–**i**) High-power transmission electron microscope images of Cu_2_Se NCs. (**j**–**l**) High-power transmission electron microscope images of Ni(OH)_2_@Cu_2_Se HHSs high magnification transmission electron microscopy images. Reproduced from ref. [15] with permission. Copyright 2023 Elsevier.

Figure 5a shows a schematic diagram of the formation of Ni(OH)_2_@Cu_2_Se hollow heterostructures, which can be seen to form a heterojunction surface. It is demonstrated in subsequent measurements that the heterostructure resulted in enhanced adsorption of glucose. The structures of glucose adsorbed on Ni(OH)_2_ and Cu_2_Se are shown in Figure 5b,c, respectively. Based on this, DFT calculations were performed to determine the glucose adsorption energies on Ni(OH)_2_ and Cu_2_Se. Comparing the glucose adsorption energy on Cu_2_Se (−1.84 eV) with that on Ni(OH)_2_ (−0.18 eV) indicates that Cu_2_Se exhibits superior adsorption performance for glucose.

The research group analyzed the morphology of Cu_2_Se NCs, Ni(OH)_2_ NCs, and Ni(OH)_2_@Cu_2_Se HHSs by high magnification transmission electron microscopy, and finally observed the morphology of Ni(OH)_2_ NCs as very small nanoparticles composed of a regular hollow cube (Figure 5d–f). The hollow structure played a crucial role for selenium infiltration. The surface of Cu_2_Se NCs consists of overlapping stacked nanorods and remains hollow (Figure 5g–i). The morphology of the final synthesized Ni(OH)_2_@Cu_2_Se HHSs is similar to that of Cu_2_Se NCs, which also consists of stacked nanorods but exhibits porous reticulated surfaces (j–l). The morphology of Ni(OH)_2_@Cu_2_Se HHSs strongly suggests that Ni(OH)_2_ NCs are encapsulated within the Cu_2_Se layer, and the above data demonstrate the structural and functional advantages of Ni(OH)_2_@Cu_2_Se HHSs in the adsorption of glucose and their significant role in food monitoring. The study is highly creative and sets a precedent for future research in this area.

It is not difficult to see that the template-directed method has unique advantages and wide applications in the synthesis of Cu_2_Se-based materials by selecting suitable templates and continuously optimizing the synthesis process to prepare Cu_2_Se-based materials with specific morphology.

### 2.2. Chemical Reduction

**Figure 6:**
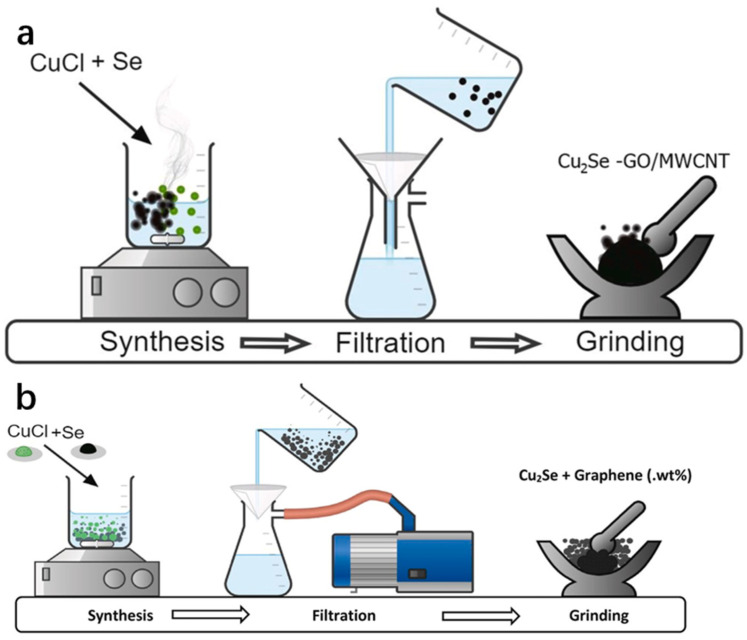
(**a**) Schematic of the synthesis process: Cu_2_Se and GO/MWCNT nanocomposites. Reproduced from ref. [16] with permission. Copyright 2023 ScienceDirect. (**b**) Schematic of the synthesis process: Cu_2_Se and Cu_2_Se-graphene nanocomposites. Reproduced from ref. [17] with permission. Copyright 2021 Elsevier.

Chemical reduction is a method of obtaining the desired product by gaining or losing electrons using a chemical reagent, often through a strong reducing agent. This method is simple to operate and does not require expensive equipment or tedious steps, but because the reaction is not carried out in a vacuum-tight environment, new impurities that are difficult to remove may be introduced during the synthesis. Sankar et al. [16] prepared Cu_2_Se powder by using the chemical reduction method and subsequently synthesized Cu_2_Se-GO (graphene oxide)/MWCNT (multi-walled carbon nanotubes) nanocomposites by using the mechanical milling method. The team firstly took ethylene glycol and water mixed in a beaker as solvent according to the volume ratio of 3:7, and then added CuCl powder and Se powder according to the stoichiometric ratio of Cu_2_Se, and stirred them well on a magnetic stirrer, keeping the temperature of the hot plate at 100 °C. Then, NaBH4 and NaOH were added and mixed well. After the reaction was complete, the product was filtered with distilled water and ethanol solution, and then dried for 3 h at 100 °C to obtain the Cu_2_Se powder. Ethylene glycol can be used as a wetting agent in the reaction; in addition, NaBH_4_ as a reducing agent, has a strong reducing property, and high temperature can accelerate the reaction rate and promote the rapid synthesis of Cu_2_Se. Subsequently, the research group mixed and milled the obtained Cu_2_Se powder with GO/MWCNT powder, which was fully milled into round particles and then sealed in Borosil tubes. The product was then placed in the furnace with a high temperature of 600 °C sintering for 2 h and finally obtained the Cu_2_Se-GO/MWCNT nanocomposites, and the specific synthesis of the process flow diagram is shown in Figure 6a. The preparation of Cu_2_Se/graphene nanocomposites was carried out by the same group, Rapaka et al. [17], using the same method, and the process flow diagram is shown in Figure 6b. Furthermore, the working group also subsequently tested the Cu_2_Se-GO/MWCNT nanocomposites and the Cu_2_Se/graphene nanocomposites and found that both of them have excellent thermoelectric properties.

Compared with the template-directed method, the chemical reduction method does not require expensive template materials, requires lower costs, and can have a certain improvement in the purity of the product through the optimization of the reaction conditions. However, since it is carried out in the laboratory, attention must be paid to safety.

### 2.3. Solid-Phase Synthesis

As a mature synthesis process, the solid-phase method is often used for the synthesis of powders, which has a wide range of applications in the preparation of many materials. It has many advantages, such as having a large yield of the prepared powder, basically no agglomeration phenomenon, good filler, and low production cost. Furthermore, the simple technology is easy to master. Until now, the solid-phase method is still the most commonly used method for powder synthesis. Two common solid-phase synthesis methods are mechanical alloying-sintering and melt synthesis methods.

**Figure 7:**
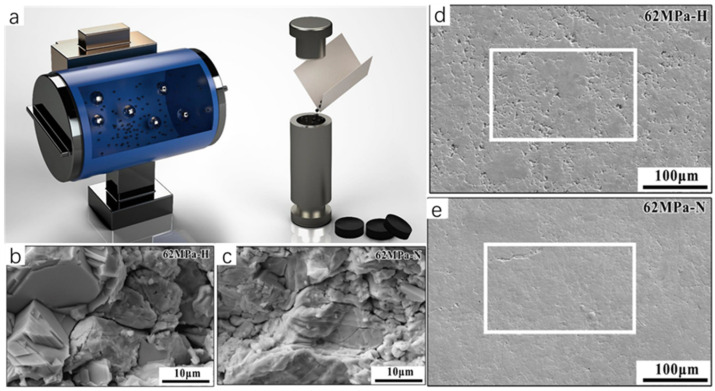
(**a**) Schematic diagram of low-temperature grinding and sintering. Reproduced from ref. [21] with permission. Copyright 2018 American Chemical Society. (**b**) SEM image of fracture morphology of Cu_2_Se sintered in 62 MPa H_2_; (**c**) SEM image of fracture morphology of Cu_2_Se sintered in 62 MPa N_2_; (**d**) SEM image of surface morphology of Cu_2_Se sintered in 62 MPa H_2_; (**e**) surface morphology of Cu_2_Se sintered in 62 MPa N_2_ SEM image. Reproduced from ref. [18] with permission. Copyright 2021 Elsevier.

The mechanical alloying–sintering method is often accompanied by high temperature and high pressure conditions, which is generally divided into the following steps: firstly, the reactants are weighed, mixed, and grounded according to a certain ratio, and secondly, the mixture is heated and pressurized in the equipment to produce a solid-phase reaction, and then the product is milled to obtain ultrafine powder (Figure 7a). In order to facilitate testing or to improve the properties of the synthesized powders, this is often accompanied by pressing the synthesized powders into flakes and subsequently sintering the samples. The melting method generally uses the raw material powder as a precursor in accordance with a certain reaction ratio in a quartz glass tube, and passes into the protective gas. The role of the protective gas is to prevent the raw materials in the reaction process and the air in the reaction of the material to produce impurities. Then, the subsequent control of the reaction temperature in the vacuum-sealed heating furnace creates the melting reaction of the raw material powder. The arc melting method, as a type of melting method, generally consists of the following operating steps. The raw material powder is first placed in a mold of the same shape as the pre-made sample and a certain amount of pressure is applied in order to compact the powder and obtain a block sample of a certain shape. This generates an arc between the electrodes or between the melted material and the electrodes to generate heat to melt the metal; the power supply is then cut off to cool to obtain a block sample. The arc melting method is easy to operate and does not require expensive equipment, complex processes, or long reaction times. It also does not require any subsequent sintering, which has attracted much attention compared to the higher temperatures required for the sintering of Cu_2_Se prepared by SPS and HP. In recent years, there are many examples of Cu_2_Se synthesized by solid-phase synthesis. For example, Sun et al. [18] used a mechanical pulverization method to directly grind and tablet purchased Cu_2_Se powders. They subsequently obtained high-density Cu_2_Se bulk materials with excellent thermoelectric properties by sintering them under high pressure in an atmosphere of hydrogen or nitrogen, and N_2_ densified the surface of Cu_2_Se (Figure 7e). Mac et al. [19] prepared Cu_2_Se with excellent thermoelectric properties by a mechanical alloying–sintering method. The Cu and Se powders were weighed and loaded into a high-energy ball mill at 700 rpm for 10 h according to the stoichiometric ratio of Cu_2_Se. The powders were subsequently pressed at 523 K and later removed and sintered at different temperatures to obtain a series of Cu_2_Se bulk samples. In addition, Salam [20] et al. successfully synthesized the Cu_2_Se thermoelectric material with excellent thermoelectric properties by using the arc melting method, reaching a ZT value of 1.46 at 873 K.

Although the solid-phase synthesis method has many advantages, it also has unavoidable disadvantages, such as low reaction efficiency, high energy consumption, and large powder particles; and impurities are often mixed in the preparation process.

### 2.4. Hydrothermal/Solvothermal Method

#### 2.4.1. Hydrothermal

Hydrothermal method has a long history as a well-established synthesis method. The hydrothermal method generally involves adding surfactant to a high temperature and high pressure aqueous solution and placing it in a reaction kettle (closed environment) to dissolve those insoluble or difficult-to-melt substances. Then, the crystals are precipitated out through the formation of a supersaturated solution by controlling the temperature difference in the reaction kettle. As a mature synthesis method, the hydrothermal method has many advantages, which includes the reaction process not requiring sintering, which can avoid the overgrowth of the crystal and the mixing of impurities. The synthesized particles are well-dispersed, have high purity, do not need high cost, and the microscopic morphology of the particles is good and controllable. However, the hydrothermal method also has the following unavoidable drawbacks: the growth process of the sample cannot be visually observed as it is conducted in a closed environment; the equipment has certain requirements (corrosion-resistant, high temperature, and high pressure); and there are certain technical requirements, such as the need for strict control of the temperature and pressure.

**Figure 8:**
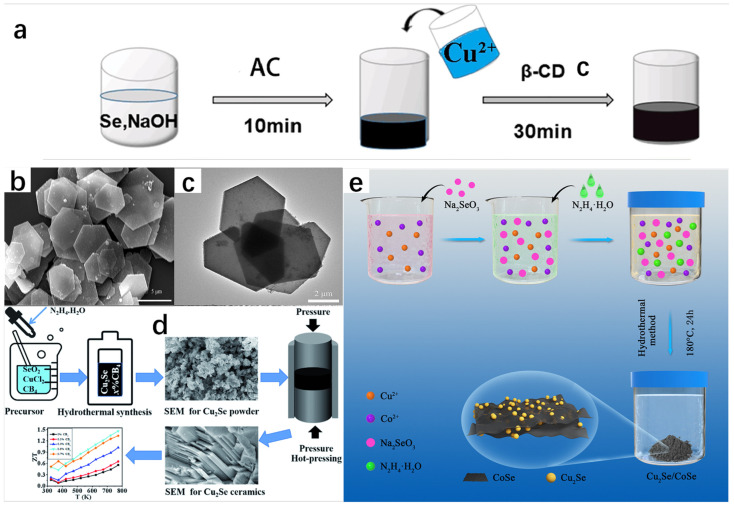
(**a**) Schematic of the synthesis of Cu_2_Se hexagonal sheets (HSs). (**b**) Typical SEM image of Cu_2_Se HSs. (**c**) Typical TEM image of Cu_2_Se HSs. Reproduced from ref. [24] with permission. Copyright 2021 Elsevier. (**d**) Flowchart, SEM image, and ZT diagram of the preparation of the Cu_2_Se+x wt%CB_4_ composite. Reproduced from ref. [25] with permission. Copyright 2022 Royal Society of Chemistry. (**e**) Flowchart for the preparation of Cu_2_Se/CoSe composites. Reproduced from ref. [26] with permission. Copyright 2021 Elsevier.

According to the relevant literature, the hydrothermal method has had many successful examples in the synthesis of Cu_2_Se and has synthesized many Cu_2_Se with different morphologies [21,22]. Dai et al. [23] successfully prepared Cu_2_Se materials using a hydrothermal method using CuCl_2_·H_2_O and Se as raw materials by adding them to a beaker containing ethylenediamine (EDA), mixing them well and then placing them in an autoclave maintained at 200 °C, and reacting them fully. Yang et al. [24] successfully prepared Cu_2_Se hexagonal sheets (HSs) using the hydrothermal method under an alkaline environment. In the synthesis, Se powder and NaOH were dissolved in a beaker in which distilled water was placed, and in order to make the dissolution as complete as possible, the beaker was placed on a magnetic stirrer and kept at 50 °C until the powder was completely dissolved. Immediately afterwards, ascorbic acid (AC), which is extremely reducing as a reducing agent, was added to the solution. Another beaker was taken and Cu(CH_3_COO)_2_ and β-cyclodextrin (β-CD) were mixed in the beaker in which distilled water was placed and stirred thoroughly. β-CD is a surfactant that increases the solubility of Cu(CH_3_COO)_2_ and reduces the surface tension of the liquid, making it easier for the liquid to penetrate the surface of the solid. Finally, the solution from the first beaker was added to the second beaker and it was observed that the mixture slowly changed from black to black-green. The black-green mixture was stirred at 40 °C for 30 min and subjected to hydrothermal treatment in a stainless-steel autoclave at 180 °C for a period of time. Then, it was cooled to room temperature, removed, and placed in a centrifuge for sufficient centrifugation. After that, it was washed repeatedly with anhydrous ethanol and distilled water. Finally, the precipitate was dried in a vacuum oven. A specific schematic diagram is shown in Figure 8a. The chemical equations involved in the reaction are as follows:

3Se + 6OH^−^ → 2Se^2−^ + SeO_3_^2−^ + 3H_2_O (4)

13SeO_3_^2−^ + 13C_6_H_8_O_6_ → 13Se + 36CO_2_ + 6H_2_O + 26OH^−^ (5)

20Cu^2+^ + C_6_H_8_O_6_ + 20OH^−^ → 20Cu^+^ + 6CO_2_ + 20H_2_O (6)

2Cu^+^ + Se^2−^ → Cu_2_Se (7)

XRD spectrum showed the successful synthesis of Cu_2_Se and the high purity of the sample. SEM image (Figure 8b) and TEM image (Figure 8c) show that the morphology of Cu_2_Se is a homogeneous hexagonal flake, with an edge length of 2~3 μm and a thickness of only 50 nm.

On the same principle, Xie et al. [25] synthesized Cu_2_Se+x wt%CB_4_ composites using the hydrothermal method and the hot pressing technique using copper chloride dehydrate (CuCl_2_·2H_2_O) and selenium dioxide (SeO_2_) as raw materials; hydrazine hydrate (N_2_H_4_·H_2_O) was used as a reductant, and boron carbide (CB_4_) nanopowders were added with different mass ratios (x = 0, 0.1, 0.3, 0.5, 0.7). CB_4_ is thermally stable and has superior electrical conductivity and Seebeck coefficient at high temperatures. These materials were added in a beaker containing deionized water and stirred in a magnetic stirrer until completely dissolved. After the solution was loaded into a stainless-steel autoclave and sealed for high-temperature heating for a period of time, it was cooled to room temperature and taken out. The sample was washed several times with ethanol and deionized water in a centrifuge and then placed in a vacuum drying oven for drying. The composite materials were then removed for vacuum sintering to obtain the dense Cu_2_Se bulk samples. The SEM morphology tests on the precursor of the Cu_2_Se nanopowder and the bulk Cu_2_Se revealed that the morphology of the precursor of the Cu_2_Se powder is an aggregated nanoplatelet, and the bulk sample is a laminated structure, which effectively enhances phonon scattering and reduces thermal conductivity, ultimately leading to the excellent thermoelectric performance of the Cu_2_Se+x wt%CB_4_ composites at 773 K. The ZT value also reached 1.46.

In addition to this, Zhu et al. [26] synthesized Cu_2_Se/CoSe composites using a hydrothermal method by placing Co(NO_3_)_2_·6H_2_O, Cu(NO_3_)_2_·3H_2_O, and distilled water in a hydrothermal autoclave and stirring well. This was followed by the addition of Na_2_SeO_3_ as a source of selenium, which was added to the autoclave and stirred well. Then, (N_2_H_4_·H_2_O) was added as a reducing agent, stirred well, and dried in an oven. This was followed by centrifugal washing with anhydrous ethanol and water for several times, and then freeze-dying to obtain Cu_2_Se/CoSe composites. The flow chart for the preparation of Cu_2_Se/CoSe composites is shown in Figure 8e. The chemical equations that occurred are as follows:

2SeO_3_^2−^ + 3N_2_H_4_·H_2_O → 2Se^2−^ + 3N_2_ + 9H_2_O (8)

4Cu^2+^ + N_2_H_4_·H_2_O → 4Cu^+^ + 4H^+^ + N_2_ + H_2_O (9)

2Cu^+^ + 2Co^2+^ + 3Se^2−^ → Cu_2_Se + 2CoSe (10)

Hydrothermal methods are also widely used in the preparation of heterojunction materials. Generally, the intermediates of the materials are first prepared by hydrothermal method, and then the heterojunction materials are obtained by a series of means.

**Figure 9:**
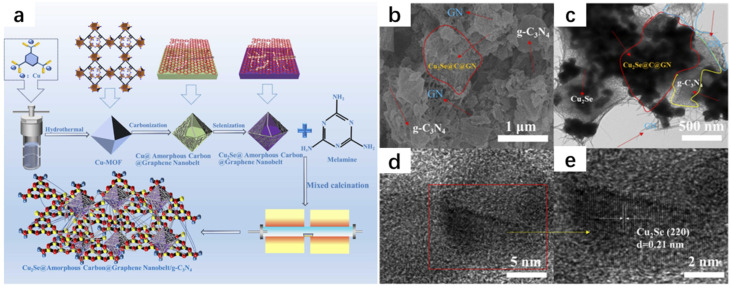
(**a**) Schematic of the synthesis process of Cu_2_Se@C@GN/g-CN. FESEM images of Cu_2_Se@C@GN/g-CN (**b**); TEM and HRTEM images of Cu_2_Se@C@GN/g-CN (**c**–**e**). Reproduced from ref. [32] with permission. Copyright 2024 Elsevier.

With the rapid development of human society, we are faced with the problem of the energy crisis. In the past, development was achieved at the expense of the environment, and in recent years, people have introduced the concept of carbon neutrality and accelerated the pace of searching for green energy, and hydrogen happens to be a type of green energy that is easy to be transported and stored [27,28,29]. There is an urgent need for a heterojunction material with high photocatalytic activity [30,31]. Liu et al. [32] successfully synthesized Cu_2_Se@Amorphous Carbon@Graphene Nanobelt/g-C_3_N_4_ (Cu_2_Se@C@GN/g-CN) composites (Figure 9a) by using the hydrothermal method and calcined annealing. The synthesis of Cu-MOF was first carried out by the hydrothermal method; 1,3,5-benzenetricarboxylic acid (C_9_H_6_O_6_) and copper nitrate hexahydrate (Cu(NO_3_)_2_·6H_2_O) were put into a beaker and added to the configured solution (N,N-dimethylformamide, ethanol, and water were mixed according to a certain ratio). In order to ensure sufficient mixing, the solution was subjected to ultrasonic stirring. The surfactant PVP was added for ultrasonic stirring and the solution was taken out and placed in an autoclave reactor, followed by drying, washing, and drying to obtain the Cu-MOF powder. The powder was then carbonized and selenized to obtain Cu_2_Se@Amorphous Carbon@Graphene Nanobelt powder, which was then reacted with melamine to obtain the Cu_2_Se@Amorphous Carbon@Graphene Nanobelt/g-C_3_N_4_ (Cu_2_Se@C@GN/g-CN) composite. The morphology was analyzed (Figure 9b–e). The photocatalytic hydrogen precipitation activity of the composite was extremely high, exceeding most of the currently known photocatalysts, and its photocatalytic properties were almost unaffected after 24 h of continuous light irradiation due to the creation of an efficiently oriented electronic channel among 2D g-C_3_N_4_ and 3D Cu_2_Se. This study is of great significance for the protection of the environment and the development of green energy.

**Figure 10:**
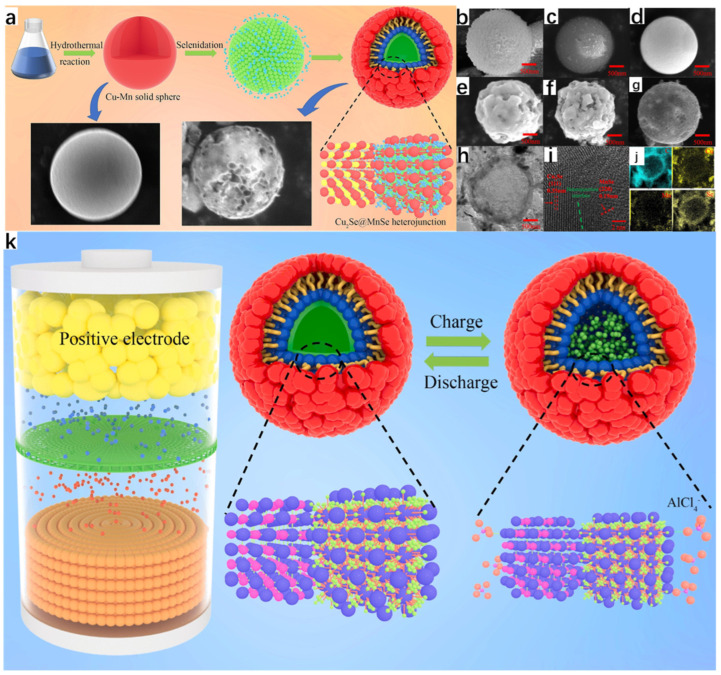
(**a**) Schematic diagram of the preparation of Cu_2_Se@MnSe heterojunction hollow spherical shells. (**b**) SEM images of Cu solid spheres. (**c**) SEM images of Mn solid spheres. (**d**) SEM images of Cu-Mn solid spheres. (**e**) SEM images of Cu_2_Se solid spheres. (**f**) SEM images of MnSe solid spheres. (**g**) SEM images of Cu_2_Se@MnSe heterojunction spherical shells. (**h**) TEM image of Cu_2_Se@MnSe heterojunction spherical shell. (**i**) HR-TEM image of Cu_2_Se@MnSe heterojunction spherical shell. (**j**) Elemental mapping image of Cu_2_Se@MnSe heterojunction. (**k**) Reaction schematic of Cu_2_Se@MnSe heterojunction spherical shell. Reproduced from ref. [33] with permission. Copyright 2023 Elsevier.

Zhang et al. [33] successfully synthesized new Cu_2_Se@MnSe heterojunction hollow spherical shells with excellent properties through a series of processes using Cu(NO_3_)_2_·3H_2_O and Mn(NO_3_)_2_·4H_2_O as raw materials, and using hydrothermal and melt-diffusion methods, which can be used as cathode materials for aluminum-ion batteries because the hydrothermal method has the advantage of synthesizing a regular morphology, so the final Cu-Mn solid spheres obtained are regular (Figure 10a). As seen from the SEM images, Figure 10b shows the Cu solid spheres; Figure 10c shows the Mn solid spheres, which have a relatively rough surface with a large number of particles attached; and Figure 10d shows the Cu-Mn solid spheres, which have a smooth surface and all three of them are relatively homogeneous in morphology. The subsequent Cu_2_Se spheres, MnSe spheres, and Cu_2_Se@MnSe heterojunction spherical shells were obtained by selenization of all three, and the Cu_2_Se@MnSe heterojunction spherical shells were obtained by selenization of Cu_2_Se@MnSe heterojunction spherical shells. MnSe heterojunction spheres have a rougher surface, which increases the contact area between the electrolyte and the spheres, resulting in a more rapid and efficient reaction. Figure 10g shows the TEM image of the Cu_2_Se@MnSe heterojunction spherical shell; it is obvious that it is a hollow structure, which prevents the volume expansion of the material in the reactant. From the lattice stripes in Figure 10i, it can be seen that the Cu_2_Se@MnSe heterojunction has been formed; Figure 10j is the EDS image, from which it can be seen that the elements of Mn, Cu, and Se are uniformly distributed, and the appearance of the C element is presumed to be because of the carbon in the spherical shell. Figure 10k shows the reaction schematic of Cu_2_Se@MnSe heterojunction spherical shells with

Charge:

Cu_2_Se + nAlCl_4_^−^ → CuSe[AlCl_4_^−^]_n_ + ne^−^ (11)

MnSe + nAlCl_4_^−^ → MnSe[AlCl_4_^−^]_n_ + ne^−^ (12)

Discharge:

4Al_2_Cl_7_^−^ + 3e^−^ → 7AlCl_4_^−^ + Al (13)

#### 2.4.2. Solvothermal Method

**Figure 11:**
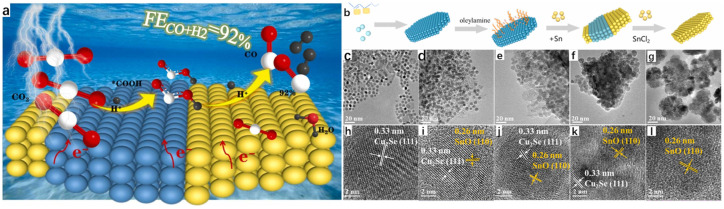
(**a**) Structural diagram showing unique 2D nanosheet structure of Cu_2_Se-SnO_0.04_ catalyst exhibiting efficient Faraday efficiency (FE). (**b**) Synthesis of Cu_2_Se-SnO heterojunction catalyst. (**c**) TEM image of Cu_2_Se. (**d**) TEM image of Cu_2_Se-SnO_0.02_. (**e**) TEM image of Cu_2_Se-SnO_0.04_. (**f**) TEM image of Cu_2_Se-SnO_0.075_. (**g**) TEM image of SnO. (**h**) HR-TEM image of Cu_2_Se. (**i**) HR-TEM image of Cu_2_Se-SnO_0.02_. (**j**) HR-TEM image of Cu_2_Se-SnO_0.04_. (**k**) HR-TEM image of Cu_2_Se-SnO_0.075._ (**l**) HR-TEM images of SnO. Reproduced from ref. [38] with permission. Copyright 2024 Elsevier.

The solvothermal method is in the process of further developing the hydrothermal method, and its process as well as its principle is more or less the same; the difference is that the solvent is no longer water, but organic matter. The electrochemical reduction of CO_2_ is a well-established method to reduce CO_2_ emissions to combat environmental pollution and to convert it into usable and efficient chemical fuels [34,35,36,37]. However, due to a number of reasons, the Faraday efficiency (FE) of the CO_2_ reduction reaction is still low. To solve this problem, Wang et al. [38] successfully synthesized two-phase Cu_2_Se-SnO heterojunction catalysts on the same plane by using the hot solvent method and the high temperature thermal reduction method, which is excellent in the selection of syngas, and the Faraday efficiency (FE) once exceeded 60% at various potentials. The team first carried out the synthesis of Cu_2_Se hexagonal nanosheets by the hot solvent method; the specific experimental procedure was to mix Se powder with oleylamine (OLAM) and 1-dodecanethiol (DDT), with OLAM as a surfactant and DDT as a reducing agent, and by keeping it at 50 °C for a certain period of time in a vacuum until the DDT dissolves Se powder completely to obtain the brown-colored solution A, which is then cooled to room temperature and filled with nitrogen. Copper acetate monohydrate (Cu(CO_2_CH_3_)_2_·H_2_O), DDT, and OLAM were mixed, degassed, stirred, and then heated with nitrogen until CO_2_CH_3_)_2_·H_2_O was completely dissolved to obtain the yellow-orange solution B. A syringe was used to inject solution A into solution B, and it was observed that the solution rapidly changed from yellow-orange to dark green. The dark green solution was treated at 220 °C for a period of time and then cooled to room temperature, and then washed and precipitated with methanol for several times to obtain Cu_2_Se nanosheets, which were subsequently synthesized by a high-temperature reduction method through a series of steps to synthesize Cu_2_Se-SnO heterojunctions, and the specific synthesis of Cu_2_Se-SnO heterojunction catalysts is shown in the schematic diagram in Figure 11b. The team analyzed and compared the microscopic morphology of the Cu_2_Se-SnO heterojunction catalyst, Cu_2_Se nanosheets, and SnO nanosheets. Transmission electron microscopy showed that Cu_2_Se is a uniform cubic nanosheet (Figure 11c), and the morphology of Cu_2_Se-SnO heterojunction catalyst behaves similarly to that of Cu_2_Se, which is still a uniform hexagonal nanosheet (Figure 11d–f), whereas SnO nanosheets are more heavily stacked. From the high-resolution transmission electron microscopy (HR-TEM), it can be seen that the Cu_2_Se-SnO heterojunction catalyst contains 0.33 nm planar row spacing value with 0.26 nm lattice spacing, which corresponds to the (111) planes of the hexagonal Cu_2_Se and the (110) planes of SnO, respectively, which successfully proves the synthesis of Cu_2_Se-SnO heterojunction catalyst; and this point is of course evident from the XRD spectrum which can also be illustrated. Among them, Cu_2_Se-SnO_0.04_ has a larger contact surface between Cu_2_Se and SnO, indicating that it has more heterojunction interfaces, and it is not difficult to see that Cu_2_Se-SnO_0.04_ has a 2D nanosheet structure in the EDX diagram. The team subsequently conducted a series of electrochemical tests, and the results showed that the Cu_2_Se-SnO_0.04_ catalyst had the best stability and selectivity in synthesis gas, and its unique 2D nanosheet structure led to a much higher rate of charge transfer, and more heterojunction interfaces led to a substantial enhancement of the charge distribution on the surface of the catalyst, which greatly improved the Faraday efficiency (FE), which reaches an amazing 92% at −0.97 V vs. RHE.

### 2.5. Ion Exchange Method

The ion exchange method is an equivalent and reversible chemical reaction carried out by ions in the liquid phase or ions in the solid phase. As a common material synthesis method, the ion exchange method can retain the morphology of the precursor well and does not require expensive equipment. The process is simple, easy to operate, causes less pollution, and has been widely used in the preparation of many materials. However, the ion exchange method also has the following limitations: in the synthesis process, it may produce impurities that are difficult to remove; and the synthesis cycle is relatively long. In the following, we will mainly introduce the cation exchange method and anion exchange method.

#### 2.5.1. Cation Exchange

**Figure 12:**
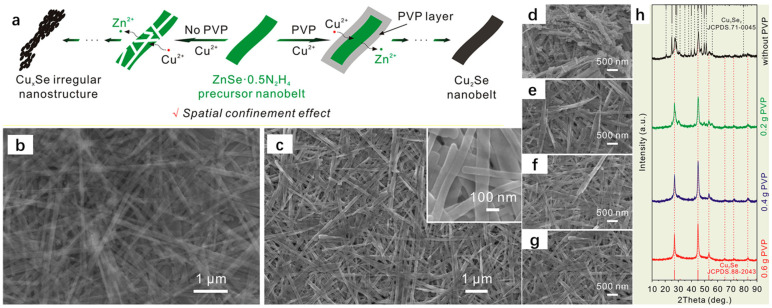
(**a**) Effect of the absence or presence of PVP on the synthesizer. (**b**) SEM images of ZnSe-0.5N_2_H_4_ nanoribbons. (**c**) SEM images of Cu_2_Se. (**d**–**g**) SEM images of PVP generators with the addition of 0 g, 0.2 g, 0.4 g, and 0.6 g. (**h**) XRD spectrum of PVP generators with the addition of 0 g, 0.2 g, 0.4 g, and 0.6 g. Reproduced from ref. [43] with permission. Copyright 2018 American Chemical Society.

Cation exchange is a common method for material synthesis. Yuan et al. [39] successfully synthesized hollow CuZnS nanoboxes (CuZnS NBs) with abundant Zn-S bonding electronic bridges at the interface by cation exchange, which greatly improved the rate of photocatalytic hydrogen precipitation, which at one time reached an astonishing 2.06 mmolg^−1^h^−1^. Yoon et al. [40] enabled the zeolite adsorbent to substantially improve its thermal storage performance by cation exchange, which was 120% of the original one. Xie et al. [41] achieved an innovative breakthrough in the preparation of Co-BiOBr by cation exchange, which greatly improved the electron–hole separation and light absorption efficiencies, with Co-BiBOR-0.5’s CO yield even reaching 11.71 μmolg^−1^h^−1^, which substantially exceeded the previous CO yield. The success of these cases is sufficient to demonstrate the superiority and feasibility of the cation exchange method. The cation exchange (CE) reaction is often used by people to synthesize nanomaterials, and the interconversion between a large number of nanocrystals is carried out by cation exchange, which is essentially the substitution of one cation by another in an intact anionic lattice; thus, this method can retain the morphology of the material precursor very well, and it has been widely accepted by the people [42]. The cation exchange method generally consists of the preparation of the precursor of the higher K_sp_ system to prepare products with lower K_sp_, but the preparation of materials by the cation exchange method is also limited by the high or low K_sp_. The Cu_2_Se nanomaterial, as a type of copper-based nanomaterial, has received wide attention for its applications in thermoelectric conversion, sensors, etc. A number of scholars have successfully synthesized Cu_2_Se with excellent thermoelectric properties by the cation exchange method. Based on the previous report by Su et al. [43], after a series of reactions of the synthesized ZnSe-0.5N_2_H_4_ nanoribbons with PVP and Cu(NO_3_)_2_, Cu^2+^ and Zn^2+^ were ion exchanged, and Cu^2+^ was reduced to Cu+ due to the reducing effect of N_2_H_4_, and the formed Cu_2_Se nanoribbons had a similar morphology to the original nanoribbons with the encapsulation of PVP, which was attributed to the fact that the cation exchange method could well retain the morphology of the precursor and the good coalescence of PVP. According to the XRD spectrum results, Cu_2_Se was successfully synthesized in the experiments of Su et al. Figure 12b shows the SEM image of ZnSe-0.5N_2_H_4_ nanoribbons, which can be seen as a regular ribbon structure, and Figure 12c shows the SEM image of Cu_2_Se, which is almost the same as that of the ZnSe-0.5N_2_H_4_ nanoribbons, which further reflects the advantages of the cation exchange method for the preparation of materials. Figure 12d shows the SEM image of the product obtained without the addition of PVP, and it can be clearly seen that its shape is an irregular nanostructure. Figure 12e–g show the SEM images of the product with the addition of 0.2 gPVP, 0.4 gPVP, and 0.6 gPVP, respectively, and it can be seen that it is a regular band structure, which is almost the same as that of the precursor. Figure 12h shows the XRD spectrum; it can be seen that in the composition of the phase without the addition of PVP, in addition to Cu_2_Se, there is Cu_3_Se_2_, showing that the phase obtained is a complex multi-phase. With the increase in the amount of PVP added, the final phase is only left with the phase of Cu_2_Se, so it can be seen that the role of the PVP surfactant is crucial. This point is also evident in the morphology of the product in Figure 12a, where the total reaction of the preparative chemical equation is 4Cu^2+^ + N_2_H_4_ = 4Cu^+^ + N_2_ + 4H^+^.

**Figure 13:**
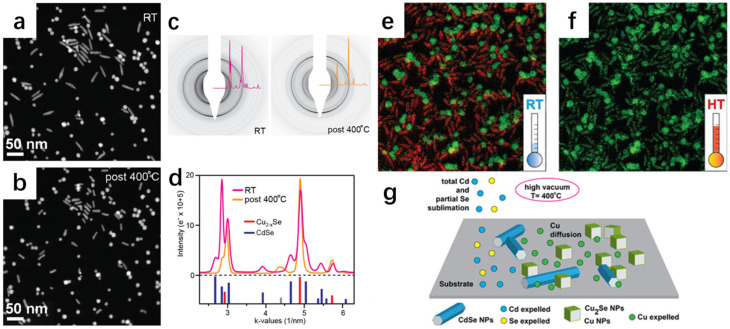
(**a**,**b**) SEM images of Cu_2_Se and CdSe NCs at room temperature versus 400 °C. (**c**) Electron diffractograms of Cu_2_Se nanorods and CdSe nanorods after heat treatment at room temperature and 400 °C superimposed on 1D profiles of ED signals obtained by integrating and by rounding off the angle in mutual inverse space. (**d**) Integrated linear spectrum of ED modes collected before 400 °C treatment and after 400 °C treatment. (**e**) Normalized energy-filtered TEM map obtained at RT. (**f**) Normalized energy-filtered TEM map obtained at 400 °C. (**g**) Schematic Diagram of Ion Exchange. Reproduced from ref. [44] with permission. Copyright 2016 American Chemical Society.

Similarly, Casu et al. [44] successfully synthesized Cu_2−x_Se nanocrystals by slow cation exchange by heating Cu_2_Se nanocrystals, Cu nanocrystals, and CdSe nanocrystals in situ on an amorphous solid substrate. Observation with transmission electron microscopy revealed that Cu_2_Se and Cu nanocrystals lost some of their Cu atoms; Cd in CdSe was completely sublimated; Se was partially sublimated; and finally CdSe was slowly converted to Cu_2−x_Se. Because it is a reaction between solids, it is relatively slow compared to that of the liquid phase, but it is precisely because of this slowness that it is convenient to propose improvements during the tracing of the experiment. From Figure 13a,b, it can be seen that there are obvious differences in the morphology before and after the annealing; before the annealing, the morphology of spherical- and rod-shaped intertwined, and the arrangement was sparse; the arrangement after annealing was more compact. Figure 13c,d show the stable coexistence of CdSe and Cu_2−x_Se before the annealing; in Figure 13e,f, the green represents the Cu atoms and the red represents the Cd atoms; the Cu atoms did not completely replace the Cd atoms; and after annealing at 400 degrees Celsius, it has been completely turned into a solid. Based on these observations, there are some proposed measures for improvement. At 400 degrees Celsius, it has completely turned into Cu_2−x_Se. Figure 13g shows the process of ion exchange. With this experience, the subsequent synthesis of other materials by the solid-state exchange method can provide a better way to observe the intermediate process and summarize the shortcomings of its synthesis as a way to improve the process.

**Figure 14:**
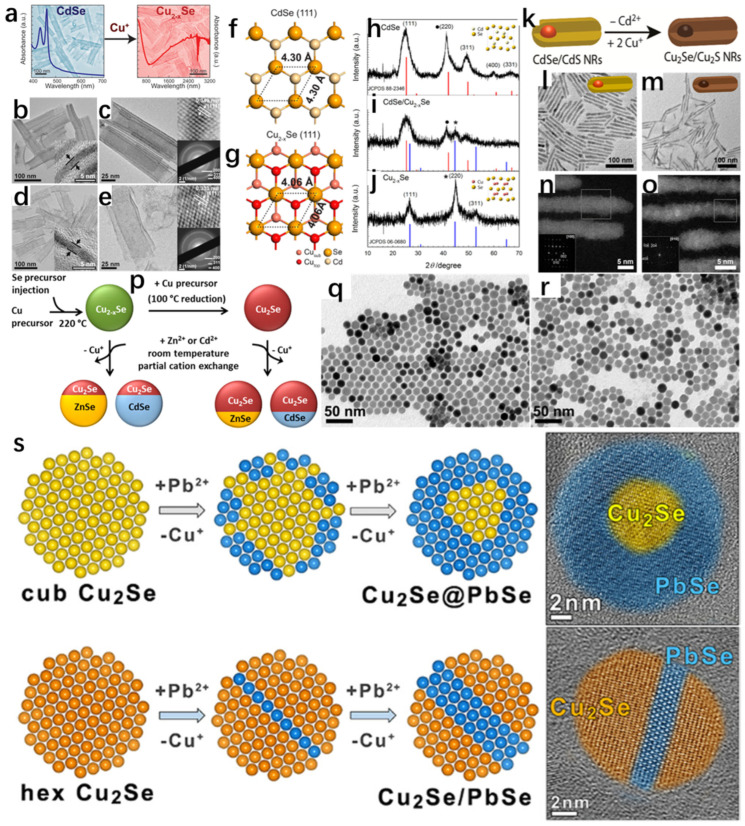
(**a**) Schematic representation of the transformation of CdSe to Cu_2−x_Se by ion exchange between Cd^2+^ and Cu^+^. (**b**) Edge TEM image of CdSe. (**c**) Top TEM image of CdSe, with the SAED image of the selected bottom region in the upper-right corner. (**d**) Edge TEM image of Cu_2−x_Se. (**e**) Top TEM image of Cu_2−x_Se, with the selected bottom SAED image of the region. (**f**) Top view of the (111) face of CdSe. (**g**) Top view of the (111) face of Cu_2−x_Se with a common anionic sublattice. (**h**) Powder XRD spectrum of CdSe. (**i**) Powder XRD spectrum of CdSe/Cu_2−x_Se. (**j**) Powder XRD spectrum of Cu_2−x_Se NSs. Reproduced from ref. [45] with permission. Copyright 2014 American Chemical Society. (**k**) CdSe/Schematic representation of the transformation of CdSNRs into Cu_2_Se/Cu_2_S NRs by ion exchange between Cd^2+^ and Cu^+^. (**l**) TEM image of CdSe/CdS NRs. (**m**) TEM image of Cu_2_Se/Cu_2_S NRs. (**n**) HRTEM image of CdSe/CdS NRs. (**o**) HRTEM image of Cu_2_Se/Cu_2_S NRs. Reproduced from ref. [46] with permission. Copyright 2015 American Chemical Society. (**p**) Cu_2−x_Se and Cu_2_Se undergo cation exchange with Zn^2+^ and Cd^2+^ to form heterojunctions at room temperature. (**q**) TEM image of Cu_2−x_SeNCs. (**r**) TEM image of Cu_2_Se. Reproduced from ref. [48] with permission. Copyright 2015 American Chemical Society. (**s**) Cation exchange reactions between cubic or hexagonal Cu_2_Se NCs and Pb^2+^ cations. Reproduced from ref. [61] with permission. Copyright 2017 American Chemical Society.

Wang et al. [45] successfully synthesized Cu_2−x_Se NSs using CdSe NSs by cation exchange (Figure 14a), which is not only extremely stable, but also has a thickness of only 1.6 nm. The cation exchange maintained the original morphology, as well as the crystal structure of pristine CdSe NSs, which is attributed to the fact that the anionic sublattice of the precursor did not change, which is effectively demonstrated in Figure 14f,g. Figure 14b,c are low–high magnification TEM images of the pristine thickness of 1.6 nm CdSe, which is seen to have a uniform rectangular shape. Figure 14d,e are low–high magnification TEM images of the thickness of 1.6 nm Cu_2−x_Se, and it can be clearly seen that there is no major difference in morphology from that of the pristine CdSe. The inset in the upper right corner of Figure 14d illustrates that the obtained Cu_2−x_Se NSs are crystalline and distinctly cubic, due to the fact that the lattice striations in the planes of cubic Cu_2−x_Se (111) preserve the cubic structure of CdSe. The XRD spectrum also demonstrates this well, owing to the fact that the cation exchange method can well preserve the sublattice structure. Bladt et al. [46] have also successfully synthesized CdSe/CdS NRs that were successfully prepared as Cu_2_Se/Cu_2_S NRs by cation exchange (Figure 14k), which also preserved the original morphology according to their TEM images.

In addition to this, in recent years, copper–sulfur carbon nanotubes have attracted widespread attention because they can maintain a large number of copper vacancies; and the more of these vacancies, the faster the rate of cation exchange [47]. Lesnyak et al. [48] used trioctylphosphine (TOP) as an exchange promoter, and the first set of Cu_2−x_Se was used in room temperature with Cu_2−x_Se and Zn(NO_3_)_2_·6H_2_O and Cd(NO_3_)_2_·4H_2_O, and an ion exchange reaction was carried out. For the second group, Cu^+^ ions were injected into Cu_2−x_Se to turn it into Cu_2_Se, and then the operation of the first group was repeated, which showed that the ion exchange rate of Cu_2−x_Se with Zn(NO_3_)_2_·6H_2_O and Cd(NO_3_)_2_·4H_2_O was faster at room temperature due to the fact that the concentration of Cu vacancies in Cu_2−x_Se was larger than the concentration of Cu vacancies in Cu_2_Se, and some of the Cu vacancies were more than the concentration of Cu vacancies in Cu_2_Se. The concentration of vacancies is larger, and some of the Cu ions are exchanged with Zn ions or Cd ions, forming the heterojunction shown in Figure 14p. The SEM images of Cu_2−x_Se and Cu_2_Se in Figure 14q,r, respectively, are seen to have retained their original sizes, shapes, and crystal structures, which are all cubic gabbro-like, after the reduction treatment. This work strongly demonstrates that the presence of Cu vacancies accelerates the rate of cation exchange in copper–selenium compounds and provides new ideas to reduce the time for cation exchange to prepare new materials in the future.

In addition to this, cation exchange (CE) reactions have been used for the synthesis of some specific structures, such as nano-heterostructures (NHs), and the synthesized materials have specific morphologies and compositions [49,50]. To better tune the morphology, structure, and properties of the materials through cation exchange reactions, an in-depth understanding of the mechanisms or kinetics of the transformations is necessary [51,52,53,54,55,56,57,58,59,60]. Gariano et al. [61] investigated the cation exchange between different phases of Cu_2_Se nanocrystals (NCs) and Pb^2+^ ions and showed that due to the different mechanisms, when the reactant is cubic Cu_2_Se, the Cu_2_Se@PbSe core@ nano-heterogeneous structure is eventually formed due to the homogeneous diffusion of Pb^2+^ ions through the Cu_2_Se crystals and low diffusion rate; when the reactant is cubic Cu_2_Se, the Pb^2+^ ions diffuse uniformly and with low diffusivity in the shell nano-heterostructure; when the reactant is hexagonal Cu_2_Se, the Pb^2+^ ions enter the (002) lattice plane and form the Cu_2_Se/PbSe-striped intermediate structure (Figure 14s). The experimental results strongly illustrate that when different phases undergo cation exchange reactions, different products are formed due to their different structures.

Above, we presented several typical examples of the successful synthesis of Cu_2_Se-based materials by the cation exchange method, and it can be found that most of the products retain the morphology of the precursors, and the reaction in the liquid phase is extremely rapid, while the reaction in the solid phase is slower. We suspect that this is because the reaction dynamics required in the solid phase is much greater than that required in the liquid phase, where the reaction proceeds from high K_sp_ to low K_sp_, whereas in the solid phase it requires high temperatures, high pressures, and many other conditions as the driving force. Later, we will introduce the anion exchange method, which is more similar to the cation exchange method.

#### 2.5.2. Anion Exchange

The anion exchange method has been widely used for the synthesis of Cu_2_Se as a common material synthesis method. Zhao et al. [62] successfully synthesized Cu_2_Se nanorods using Cu_2_O nanosheets as precursors by using the anion exchange method. They first synthesized Cu_2_O nanosheets and subsequently obtained Cu_2_Se nanorods by the exchange of Se^2−^ ions with O^2−^ ions. The successful preparation of both Cu_2_O nanosheets and Cu_2_Se nanorods was demonstrated by XRD spectrum. The possible reactions involved in the synthesis process are as follows:

Cu^2+^ + 2OH^−^ = Cu(OH)_2_↓ (14)

Cu(OH)_2_ + C_6_H_8_O_6_ = Cu_2_O + 2H_2_O + C_6_H_6_O_6_ (15)

Cu_2_O + Se^2−^ + H_2_O = 2OH^−^ + Cu_2_Se (16)

**Figure 15:**
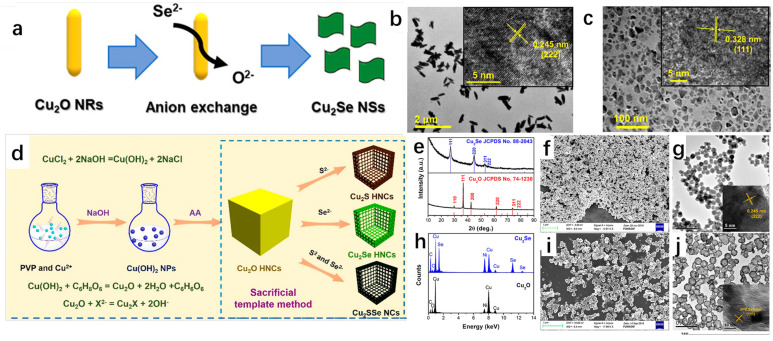
(**a**) Schematic diagram of the synthesis of Cu_2_SeNSS by Cu_2_O NRS via S^2−^ ion exchange with O^2−^ ion exchange. (**b**) TEM image of Cu_2_O. (**c**) TEM image of Cu_2_Se. Reproduced from ref. [62] with permission. Copyright 2020 American Chemical Society. (**d**) Synthesized diagram of Cu_2_X (X = S, Se, SSe). (**e**) XRD spectrum of Cu_2_Se with Cu_2_O. (**f**) SEM image of Cu_2_O NCs. (**g**) TEM image of Cu_2_O HNCs; inset is the corresponding HRTEM image. (**h**) EDS map of Cu_2_Se and Cu_2_O. (**i**) SEM image of Cu_2_Se HNCs. (**j**) TEM image of Cu_2_Se HNCs; inset is the corresponding HRTEM image. Reproduced from ref. [63] with permission. Copyright 2019 American Chemical Society.

Figure 15a shows the schematic diagram of Se^2−^ ion exchange with O^2−^ ion, which can briefly describe the synthesis process of Cu_2_Se nanorods. Figure 15b,c show the TEM images of Cu_2_O and Cu_2_Se, respectively, and it can be seen that the morphology of Cu_2_O is a rod with uniform size, while the morphology of Cu_2_Se is a denser flake. Similarly, Wang et al. [63] successfully synthesized Cu_2_Se hollow nanocubes (HNCs) by the anion exchange method using Cu_2_O nanocubes (NCs) as templates, and by changing the type of reactants. This method can also synthesize Cu_2_S HNCs or Cu_2_SSe NCs with similar structures. The schematic diagram of their synthesis is shown in Figure 15d, in which PVP acts as a capping agent and AA is ascorbic acid. It is easy to see that the morphology of the synthesized product without PVP will be heterogeneous morphology; X^2−^ ion is S^2−^ ion or Se^2−^ ion or S^2−^ and Se^2−^ ion, and the synthesized product is different according to the X^2−^. Figure 15e shows the XRD spectrum, which proves that Cu_2_O was successfully synthesized and converted to Cu_2_Se, and the purity of the synthesized Cu_2_Se is relatively high. According to the general conclusion in the literature, the reaction proceeds in the direction from the substance with large K_sp_ to the substance with small K_sp_. These two works not only successfully synthesized Cu_2_Se but also conveyed a message that the K_sp_ of Cu_2_Se is lower than that of Cu_2_O.

However, although the ion exchange method has many advantages, the ion exchange often occurs in the chemical reaction and is limited by the size of K_sp_ and the ability to control the reaction conditions accurately, etc. These factors affect the final synthesis of Cu_2_Se. These factors affect the final synthesis results.

### 2.6. Summary of Methods

In order to facilitate the reader to have a deeper understanding of the five common methods, this manuscript summarizes the advantages and disadvantages of these methods in Table 1.

## 3. Energy Conversion and Storage

### 3.1. Energy Conversion

Sulfur compounds can be used in many fields such as light-emitting diodes, photodetectors, photovoltaic cells, and supercapacitors due to their unique optical properties and catalytic activity [64,65,66,67]. As a typical sulfur genus compound, Cu_2_Se has attracted much attention for its excellent carrier mobility and significant thermoelectric figure of merit (ZT value) and shows a broad application prospect in the field of thermoelectric energy conversion. It has been shown that the thermoelectric properties of Cu_2_Se-based materials can be significantly enhanced by optimizing the synthesis process and modulating the material microstructure, thus achieving more efficient thermoelectric conversion efficiency. An in-depth discussion of the optimization strategy of the thermoelectric properties of Cu_2_Se-based materials and their intrinsic mechanism not only helps to build understanding about the nature of the thermoelectric material performance enhancement, but also lays a theoretical foundation for the development of high-performance thermoelectric devices. Given that its optimal performance occurs at higher temperatures, Cu_2_Se-based materials show broad application prospects in industrial waste heat recovery power generation systems. Research into their application in wearable devices remains in its early stages, facing challenges such as achieving near-room-temperature performance and material flexibility.

#### 3.1.1. Electronic and Phonon Transport Properties of Cu_2_Se

To achieve the design of high-performance Cu_2_Se-based thermoelectric or energy storage materials, it is essential to gain a deep understanding of their unique electronic and phonon transport behaviors and the interactions between them. The exceptional performance of Cu_2_Se, particularly its extremely high thermoelectric figure of merit (ZT), fundamentally stems from its “phonon glass-electron crystal” characteristics, which maintains high electronic conductivity, while possessing extremely low lattice thermal conductivity.

The electronic properties of Cu_2_Se are intrinsically linked to its crystal structure, most notably in the β phase (face-centered cubic structure) that remains stable at elevated temperatures. Within this phase, the Se sublattice forms a rigid framework, while Cu^+^ ions exhibit highly disordered, “quasi-liquid-like” migration behavior at interstitial sites. This mobile Cu^+^ system not only provides abundant hole carriers but also endows the material with an intrinsic conductivity approaching that of metals. Practically synthesized Cu_2_Se often exhibits non-stoichiometric compositions (Cu_2−x_Se), where the copper vacancy concentration (x) is the key factor in regulating carrier density. By precisely controlling synthesis conditions or introducing dopant elements, carrier density can be effectively optimized to balance conductivity and Seebeck coefficient, thereby achieving higher PF.

On the other hand, Cu_2_Se’s low thermal conductivity, particularly its extremely low lattice thermal conductivity, is another key factor in its outstanding thermoelectric performance. The quasi-liquid behavior of Cu^+^ ions causes strong point defect scattering of phonons, which is the primary reason for its low intrinsic thermal conductivity. This scattering mechanism originates from the dynamic interaction between lattice vibrations (phonons) and migrating copper ions. Furthermore, synthetic strategies such as nanostructuring, introducing second phases, or constructing porous microstructures can introduce additional scattering centers like grain boundaries and interfaces. This enhances scattering effects on medium-to-long-wavelength phonons, thereby further suppressing lattice thermal conductivity. The synergistic mechanism between electrons and phonons is central to achieving high performance. In Cu_2_Se, charge carriers achieve efficient transport via high mobility, while phonons undergo strong scattering due to multiscale defects. The rigid Se framework provides a stable channel for electron transport, whereas migrating Cu^+^ ions, while contributing to conductivity, also disrupt lattice vibrations like a “phonon solvent,” partially decoupling the transport pathways of electrons and phonons.

In summary, an in-depth understanding of the electronic and phonon behavior in Cu_2_Se reveals that its exceptional thermoelectric performance stems from the inherent electron–phonon decoupling properties of its crystal structure. Future material design should continue to focus on microstructural control, introducing multiscale phonon scattering mechanisms while maintaining excellent electrical transport capabilities. This will further enhance the decoupling effect, driving breakthroughs in thermoelectric performance.

#### 3.1.2. Optimization of Thermoelectric Properties

Based on the above understanding of the electronic and phonon transport properties of Cu_2_Se, researchers have developed various performance optimization strategies. It is a simple and easy way to optimize the thermoelectric properties of Cu_2_Se by using different reaction methods to control different reaction conditions. The design of specific components requires selecting appropriate synthesis methods while precisely controlling certain parameters, such as precursor design, reaction atmosphere control, and annealing process regulation. In precursor design, the ratio of Cu to Se (e.g., Cu_2.2_Se, Cu_1.9_Se) can be engineered to precisely regulate copper vacancy concentration, thereby optimizing carrier concentration. More reactive Se sources can be employed to prevent Se agglomeration or incomplete reactions. High-energy ball milling can achieve nanoscale mixing of raw materials, ensuring more uniform material reactions. Controlling the atmosphere, particularly during high-temperature synthesis (e.g., sintering, melting), is crucial. Ensuring sealing integrity and introducing a controlled atmosphere (e.g., vacuum, inert gas protection) prevents Se volatilization, thereby controlling the composition of the final product. Regarding annealing process regulation, a carefully designed annealing process can eliminate the internal stresses generated during synthesis, and promote atomic diffusion and rearrangement, thereby improving crystallinity and compositional uniformity, and stabilizing specific crystal phases. For microwave synthesis, its unique “bulk heating” characteristic inherently promotes more uniform nucleation and growth compared to traditional external heating. Simultaneously, optimizing the distribution of microwave absorbers (e.g., SiC) and reactor design can further enhance thermal field uniformity. For ball milling, controlling grain refinement and amorphization levels is achieved by optimizing milling parameters (rotation speed, ball-to-material ratio, duration), thereby avoiding impurity introduction or composition segregation caused by excessive grinding.

The thermoelectric properties of Cu_2_Se strongly depend on its microstructure, phase composition, and grain size. Therefore, selecting an appropriate synthesis method is crucial for achieving target performance. Solid-state synthesis methods (e.g., mechanical alloying-sintering) readily produce high-density bulk materials and are commonly employed to achieve high thermoelectric figure of merit (ZT). Conversely, hydrothermal/solvothermal methods facilitate precise control over nanosheet morphology, reducing thermal conductivity by enhancing phonon scattering. This section will explore how these synthesis approaches optimize thermoelectric performance by regulating material microstructure.

**Figure 16:**
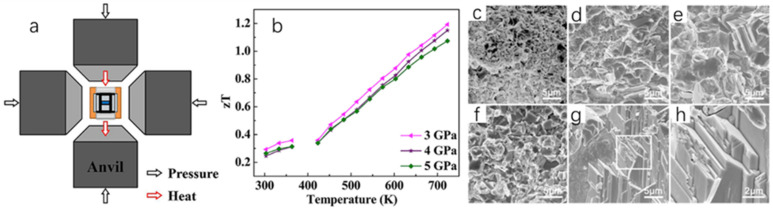
(**a**) HPHT-synthesized Cu_2_Se device. (**b**) Temperature dependence of ZT values of Cu_2_Se synthesized at different pressures. (**c**–**h**) SEM images of Cu_2_Se synthesized at different temperatures and pressures of 3 GPa: (**c**) 25 °C, (**d**) 240 °C, (**e**) 540 °C, (**f**) 840 °C, (**g**) 1000 °C, and (**h**) selected regions of 1000 °C. Reproduced from ref. [68] with permission. Copyright 2019 Elsevier.

Xue et al. [68] successfully prepared Cu_2_Se bulk samples by mechanical alloying–sintering under high temperature and high pressure (HPHT) conditions, and regulated their thermoelectric properties by controlling the pressure and temperature of the synthesis. The team first weighed a certain amount of Cu and Se and mixed them according to the stoichiometry of Cu_2_Se, and subsequently grounded the mixture. This was followed by cold pressing the powder into a disk shape and obtaining different samples by varying the pressure and temperature of the synthesis and treating them for 30 min in a device (Figure 16a). Finally, the disk-shaped samples are pulverized and milled to measure their thermoelectric properties. The increase in the temperature of the synthesis affects the microscopic morphology of the Cu2Se samples, as seen in Figure 16c–h. The microscopic morphology of the Cu2Se samples is a layered, stacked structure; and the higher the synthesis temperature, the larger the particle size of the samples, and the higher the crystallinity. This structure suppresses the lattice thermal conductivity of the sample. The increase in synthesis temperature also has an effect on the carrier concentration of Cu_2_Se, which in turn increases the power factor of the material. Lattice distortion and yield deformation occur under high pressure, which reduces the activation energy between Se and Cu atoms, and shortens the reaction time to 30 min, which greatly improves the reaction efficiency [69]. The results show that the best thermoelectric properties of the Cu_2_Se powders occur when they are prepared at 1000 °C and 3 GPa, with the ZT value of 1.19 at 723 K (Figure 16b). It is reasonable to believe that mechanical alloying–sintering under HPHT conditions is also capable of synthesizing other thermoelectric materials with excellent thermoelectric properties.

**Figure 17:**
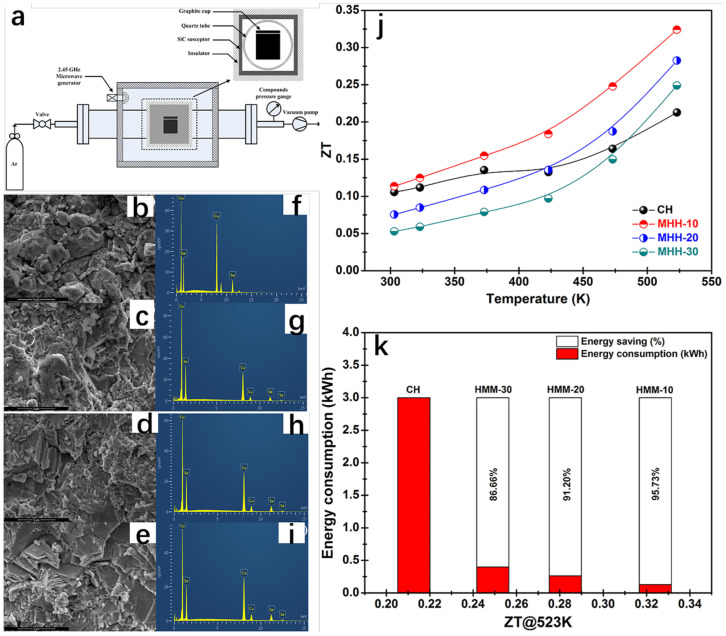
(**a**) Schematic diagram of the microwave hybrid heating device. (**b**) SEM micrographs of Cu_2_Se particulate samples fabricated by the conventional heating (CH) method. (**c**) SEM micrographs of Cu_2_Se particulate samples fabricated by the MHH-10 method. (**d**) SEM micrographs of Cu_2_Se particulate samples fabricated by the MHH-20 method. (**e**) MHH-30 method fabricated by the SEM micrographs of Cu_2_Se particulate samples. (**f**) EDS spectra of Cu_2_Se particulate samples produced by the conventional heating (CH) method. (**g**) EDS spectra of Cu_2_Se particulate samples produced by the MHH-10 method. (**h**) EDS spectra of Cu_2_Se particulate samples produced by the MHH-20 method. (**i**) The MHH-30 method produced Cu_2_Se particle samples by MHH and conventional heating (CH) methods. (**j**) The variation in the preferred value of Cu_2_Se samples synthesized by MHH and conventional heating (CH) methods with temperature. (**k**) The ZT value of Cu_2_Se samples synthesized by MHH and conventional heating (CH) methods with energy consumption at 523 K. Reproduced from ref. [75] with permission. Copyright 2021 Elsevier.

The microwave hybrid heating method, as a type of mechanical alloying–sintering method, is often used to synthesize β-Cu_2_Se. In addition to this, there are a number of other methods of synthesizing β-Cu_2_Se, such as ball milling [70], solvent-heated and hydrothermal synthesis [71,72]. The microwave hybrid heating (MHH) method requires a short period of time and less energy. It improves the powder with larger particles by heating in two directions, from the outside to the inside by using a silicon carbide sensitizer, and from the inside to the outside directly by microwave energy [73,74]. Rudradawong et al. [75] successfully synthesized Cu_2_Se thermoelectric materials dominated by β-Cu_2_Se using the microwave hybrid heating technique (MHH) and exhibited very low thermal conductivity and excellent thermoelectric properties. The Cu_2_Se thermoelectric materials were synthesized using microwave mixing and heating (MHH). The Cu and Se were weighed according to the stoichiometric ratio of 2:1 and ball-milled in a ball mill, and subsequently the mixed powders were placed in a microwave mixing and heating oven (Figure 17a) with the power maintained at 800 W, and different samples were obtained by regulating the heating time (10 min, 20 min, 30 min). In order to compare with the Cu_2_Se samples obtained by the conventional method, the mixed powder of Cu and Se was calcined at high temperature to obtain Cu_2_Se powder. The powders obtained by the conventional method and the microwave hybrid heating method were milled, pressed, sintered, and cooled to facilitate the subsequent measurement of thermoelectric properties. The ZT value of Cu_2_Se synthesized under the 10 min microwave hybrid heating technique reaches 0.32 at 523 K. We all know that Cu_2_Se is a monoclinic phase α-Cu_2_Se at low temperatures and transforms into a face-centered cubic phase β-Cu_2_Se when the temperature reaches 410 K [76]. Moreover, β-Cu_2_Se has a high concentration of Cu^+^ ions as compared to α-Cu_2_Se. These Cu^+^ ions exist in the lattice in a disordered manner, which reduces the free range of motion of phonons, and the vibrations of phonons are partially eliminated [77], resulting in a decrease in lattice thermal conductivity. The high concentration of Cu^+^ ions also gives the material a high electrical conductivity and a low Seebeck coefficient. XRD spectrum tests are conducted on Cu_2_Se samples, which are synthesized by heating for different time periods using the MHH method, and by heating for 3h using the conventional method. While only α-Cu_2_Se was observed for the Cu_2_Se synthesized by the conventional method at room temperature, a mixed phase of α-Cu_2_Se and β-Cu_2_Se was observed for the Cu_2_Se synthesized by the MHH method, and the proportion of β-Cu_2_Se increased with the increase in the MHH time. The group then performed SEM analysis (Figure 17b–e) and EDS analysis (Figure 17f–i) on the samples and found that the SEM images of the samples showed thin lamellar layers stacked on top of each other, with a very small size of just a few micrometers. The EDS spectra showed that the Cu:Se was becoming bigger and bigger, and the samples obtained by MHH were above two, which was due to the evaporation of the Se which made an overabundance of Cu. The conductivity of the samples is shown to decrease with increasing temperature, which exhibits a distinctly condensed semiconductor behavior. Simplified semiconductors are impurity semiconductors with a high doping concentration, which are close to the properties of metals. The conductivity of metals decreases with increasing temperature because the formation of an electric current is the result of the directional movement of electrons, and as the temperature increases, the original atoms in the metal move at a faster rate and collide with the electrons, resulting in a decrease in conductivity. And as the time of MHH processing increases, the proportion of β-Cu_2_Se increases, and the concentration of carriers becomes higher and higher, as the mobility becomes lower; this increases the carrier concentration by an order of magnitude greater than that of the carrier mobility, which becomes lower. So, the conductivity of Cu_2_Se samples increases with the increase in MHH treatment time. The Seebeck coefficient decreases as the carrier concentration rises, while the carrier concentration rises with the MHH treatment time, which can be interpreted as a decrease in the Seebeck coefficient with an increase in the MHH treatment time. Combining the Seebeck coefficient and conductivity, we can deduce that the PF of MHH-30 achieves the maximum value of 7.90 × 10^−4^ W m^−1^K^−2^ at 673 K. Analyzing the basics of thermoelectricity in conjunction with this study, it is easy to conclude that the increase in conductivity is due to an increase in carrier concentration, which is due to an increase in β-Cu_2_Se. That is, the longer the MHH treatment, the higher the carrier concentration, and the higher the conductivity, the higher the K_c_, and the higher the K. Combining the power factor PF and thermal conductivity K, in general, the Cu_2_Se sample of MHH-10 has the highest ZT value of about 0.32 at 573 K, which exceeds the 0.21 of the conventional synthesis method, and it saves 85% for energy consumption, while greatly shortening the reaction time (Figure 17j,k).

**Figure 18:**
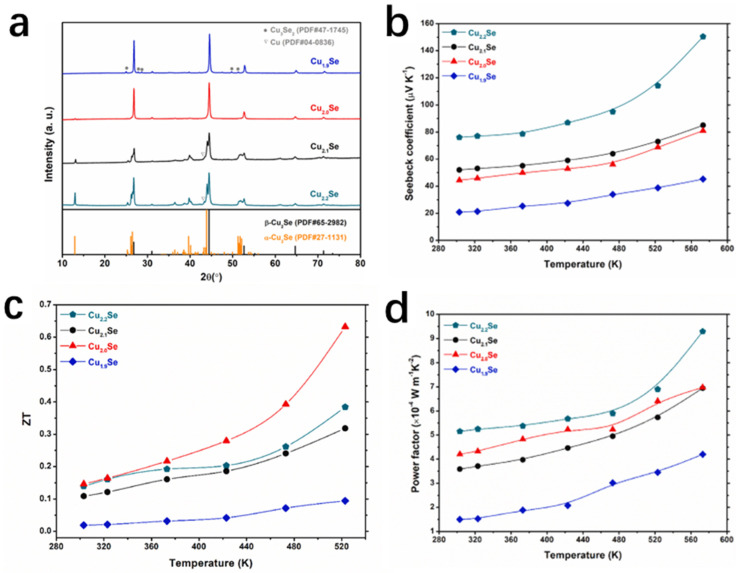
(**a**) XRD spectrum of Cu_1.9_Se, Cu_2.0_Se, Cu_2.1_Se and Cu_2.2_Se samples synthesized by the 10 min MHH method. (**b**–**d**) Temperature dependence of TE properties of Cu_2.2_Se, Cu_2.1_Se, Cu_2.0_Se, and Cu_1.9_Se samples. (**b**) Seebeck coefficient. (**c**) ZT value. (**d**) Power factor. Reproduced from ref. [78] with permission. Copyright 2024 Elsevier.

Similarly, Sakulkalavek et al. [78] synthesized β-Cu_2.0_Se with excellent thermoelectric properties by the MHH method. It is well known that the thermoelectric properties and structures of Cu_2_Se-based thermoelectric materials are inextricably linked to their stoichiometry [79,80,81]. The research group synthesized Cu_x_Se_y_ compounds with different ratios (x:y = 2.2:1.0, 2.1:1.0, 2.0:1.0, and 1.9:1.0) by controlling the stoichiometric ratios of Cu and Se, and the mixed powders were subsequently milled well, and prepared by the MHH method. The bulk sample was grounded and sieved then pressed, and finally sintered under an atmosphere of argon gas and cooled to room temperature and removed. XRD spectrum (Figure 18a) illustrates that by controlling the ratio of Cu:Se, the ratio of β-Cu_2_Se can be controlled, optimizing the carrier concentration and thermal conductivity, which ultimately results in Cu_2.2_Se reaching the maximum PF at 573 K. However, due to the excess of Cu, the thermal conductivity of Cu_2.2_Se is too high, while the thermal conductivity of Cu_2.0_Se is low, and combined with the high PF of Cu_2.0_Se, the final ZT value of Cu_2.0_Se reaches a high level of 0.65 at 523 K (Figure 18d).

**Figure 19:**
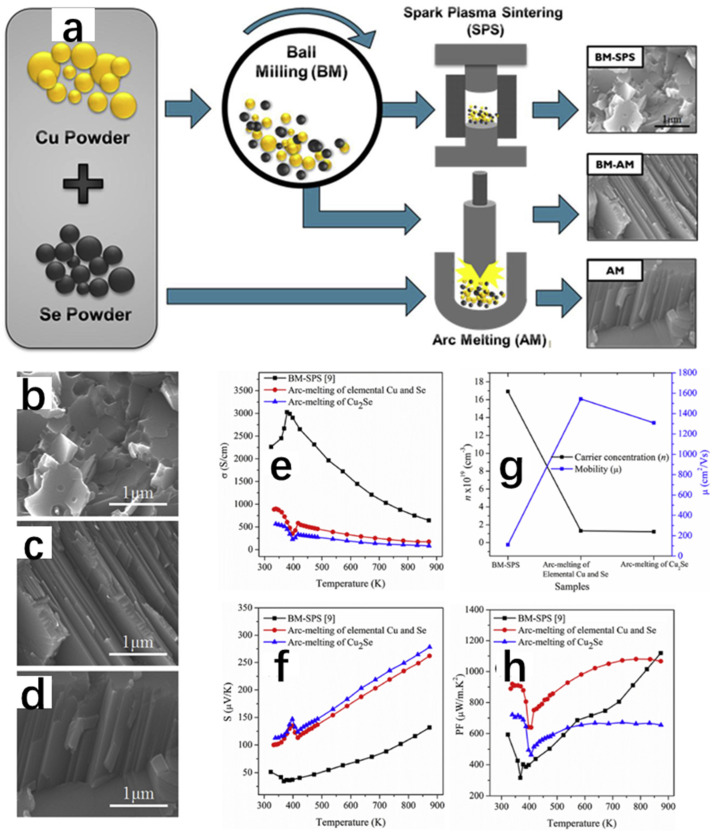
(**a**) Scanning electron micrographs of Cu_2_Se samples synthesized by three different methods: ball milling with spark plasma sintering technique, ball milling with arc melting, and arc melting. (**b**) Scanning electron microscope diagrams of Cu_2_Se samples synthesized by ball milling with spark plasma sintering technique. (**c**) Sweep electron microscope diagrams of Cu_2_Se samples synthesized by ball milling with arc melting. (**d**) Arc melting method for synthesizing Cu_2_Se samples. Scanning electron micrographs. (**e**) Variation in conductivity with temperature of Cu_2_Se samples synthesized by three different methods. (**f**) Variation in Seebeck coefficient with temperature of Cu_2_Se samples synthesized by three different methods. (**g**) Variation in carrier concentration and carrier mobility of Cu_2_Se samples synthesized by three different methods. (**h**) Variation in power factor with temperature of Cu_2_Se samples synthesized by three different methods. Reproduced from ref. [20] with permission. Copyright 2019 Elsevier.

In another work, Butt et al. [20] successfully synthesized Cu_2_Se thermoelectric materials by arc melting, and the thermoelectric properties were excellent, with a ZT value of 1.46 at 873 K. As shown in Figure 19a, the team used three methods to synthesize Cu_2_Se. The first one was to synthesize the polycrystalline Cu_2_Se powders by ball milling [82], followed by the synthesis of polycrystalline Cu_2_Se powders by sparking at a temperature of 500 °C and 50 MPa at a pressure of 500 °C. The first method was to synthesize the polycrystalline Cu_2_Se powders by ball milling with a pressure of 500 °C into solid pellets by spark plasma sintering; the second method included first obtaining polycrystalline Cu_2_Se powder by ball milling, then compressing it into a solid with a pressure of 4 MPa, and finally compressing the Cu_2_Se into pellets by arc melting; and the third method was basically the same as the second one, except that the polycrystalline Cu_2_Se powder obtained by ball milling was replaced by a stoichiometric mixture of 2Cu and Se. The three samples obtained were subjected to XRD spectrum and SEM characterization, and the results of the XRD spectrum showed that the Cu_2_Se samples synthesized by these three methods all possessed both α-Cu_2_Se and β-Cu_2_Se mixed phases at room temperature, and all of them turned into β-Cu_2_Se again at 473 K, which illustrated the transition of the Cu_2_Se crystals from the room-temperature α-Cu_2_Se to the high-temperature β-Cu_2_Se. Although the crystal phases obtained by all three methods are consistent, the third method is faster, has the lowest economic loss, and has the highest degree of crystallinity, which is not difficult to prove from the intensity of their diffraction peaks. Figure 19b shows the scanning electron microscope images of the crushed samples obtained by BM-SPS (ball-milling method with spark plasma sintering) treatment, and we can see that the samples show smaller grains and fewer pores, which may be due to the evaporation of selenium caused by too high temperatures under the SPS treatment. Furthermore, we can see some grain boundaries at the same time, which may have a detrimental impact on the transport of carriers, and consequently on the thermoelectric properties. Figure 19c,d show the SEM images of the crushed samples obtained by BM-AM (ball milling with arc melting) and AM (arc melting), respectively, and it can be seen that the grains are presented as lamellar, and the porosity is also small, which is favorable for carrier transport. Figure 19e–h show the thermoelectric properties, and the final results show that the arc melting method has successfully synthesized pure Cu_2_Se with good thermoelectric properties, which is a simple process with less loss cost, providing stable properties and a 70% increase in ZT relative to Cu_2_Se obtained by spark plasma sintering after ball milling, but we have no way of knowing how this method can be applied to the synthesis of other thermoelectric materials, and it needs to be further researched.

**Figure 20:**
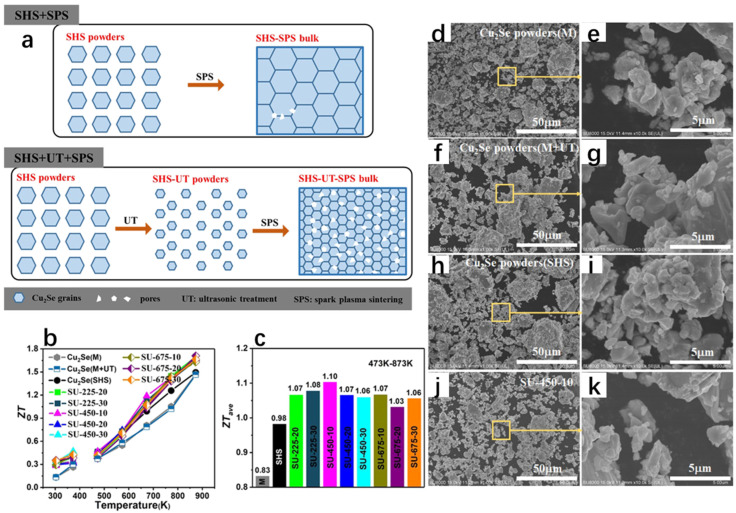
(**a**) Schematic representation of in situ hole formation in self-propagating pyrosynthesized Cu_2_Se bulk after UT and without UT. (**b**) ZT values of Cu_2_Se prepared by melting, melting + ultrasound preparation, self-propagating pyrosynthesis, and self-propagating pyrosynthesis + ultrasound, respectively. (**c**) Average ZT values of Cu_2_Se from 473 K to 873 K for different preparation methods. (**d**) FESEM image of melt-synthesized Cu_2_Se powder. (**e**) Magnified image of the yellow rectangle-marked area in (**d**). (**f**) Melt synthesized Cu_2_Se powder followed by sonication. (**g**) Magnified image of the yellow rectangle-marked area in (**f**). (**h**) Cu_2_Se powder synthesized by self-propagating high temperature synthesis only. (**i**) Magnified image of the yellow rectangularly marked area in (**h**). (**j**) Cu_2_Se powder synthesized by the self-propagating high-temperature synthesis method followed by ultrasonication (450 W for 10 min). (**k**) Magnified image of the yellow rectangularly marked area in (**j**). Reproduced from ref. [83] with permission. Copyright 2021 Elsevier.

Compared to the melt-only synthesis of Cu_2_Se, Zhang et al. [83] discovered a new path to improve the thermoelectric properties of Cu_2_Se by synthesizing Cu_2_Se materials with excellent thermoelectric properties through a combined method of self-propagating high-temperature synthesis (SHS), ultrasonic treatment (UT), and spark plasma sintering (SPS) (Figure 20a), which led to the enhancement of phonon scattering and the decrease in thermal conductivity, which was only 0.40–0.45 Wm^−1^K^−1^ at 873 K, compared to the molten samples. The ZT value was substantially increased by 16% at 873 K (Figure 20b), and further FESEM analysis was performed for the samples synthesized by different methods (Figure 20d–k), and it can be seen that the morphology of the material does not change significantly after ultrasonic treatment, and the grain size is refined, which in turn leads to enhanced phonon scattering and reduced thermal conductivity. The thermoelectric properties of SHS+SPS samples are improved by ultrasonic treatment, which is not only low cost but also very efficient, and is of great significance for the improvement in the thermoelectric properties of Cu_2_Se.

As a well-established thin film synthesis method, magnetron sputtering is the first choice for the preparation of high-performance thermoelectric thin films because of the three major advantages of accurate control of the material composition, uniform distribution of the produced films, and the simple equipment required [84,85,86,87]. The basic principle is as follows: on the surface of a solid, molecules on the solid surface are bombarded with high-energy particles (usually positive ions accelerated by an electric field); atoms and incident high-energy particles exchange kinetic energy from the solid surface, sputtering out of the atoms (or atomic groups) which has a certain energy; they can be re-coalesced on the surface of the solid substrate to form a thin film. The magnetron sputtering method changes the ratio of sputtering elements and the substrate temperature to achieve the control of carrier concentration and material micro morphology, so as to regulate the Seebeck coefficient, electrical conductivity, and thermal conductivity, and improve the thermoelectric properties of materials. Magnetron sputtering has excellent applications in the synthesis of thermoelectric thin films with excellent thermoelectric properties. Lan et al. [84] doped Sn into GeTe by magnetron sputtering. The changes in the thermoelectric properties of the crystal films at low and high temperatures were investigated by varying the content of Sn. It was finally found that the Seebeck coefficient and power factor of the crystal films were enhanced due to the optimization of the carrier concentration as well as the resistivity, and the power factor reached 1.423 × 10^3^ μW/K^2^m at 718 K. In addition to this, a great deal of effort has been invested into researching thermoelectric films suitable for use at low temperatures [88,89,90,91] with great progress made on BiTe_3_, but due to its high cost, lack of raw materials, and the presence of small amounts of toxins, it was not commercially viable. People have to put their attention to other materials to replace BiTe_3_ [92,93,94,95,96]. Due to the abundance of raw materials, as well as Cu_2_Se being non-toxic and harmless, it has naturally become a substitute for BiTe_3_ [97].

**Figure 21:**
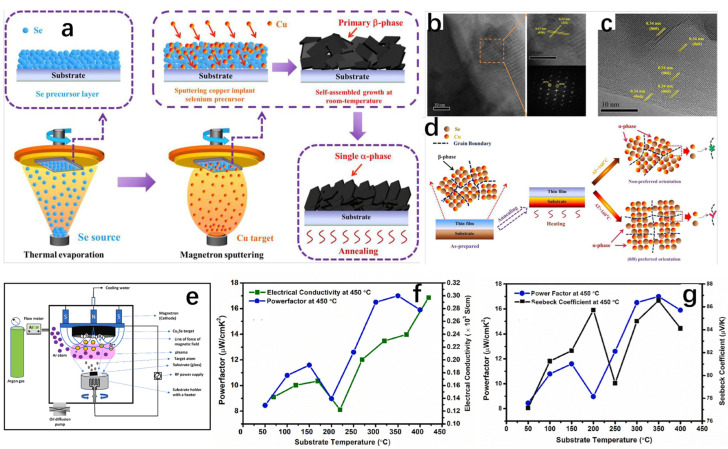
(**a**) α-Cu_2_Se thin film fabrication image. (**b**) HRTEM image of α-Cu_2_Se thin film whose annealing temperature is 160 °C. (**c**) HRTEM image of α-Cu_2_Se thin film whose annealing temperature is 240 °C. (**d**) Schematic of preferred growth orientation of α-Cu_2_Se thin films with different annealing temperatures. Reproduced from ref. [98] with permission. Copyright 2021 Elsevier. (**e**) Schematic of Cu_2_Se film deposition. (**f**) Variation in power factor and conductivity with substrate temperature at 450 °C. (**g**) Variation in power factor and Seebeck coefficient with substrate temperature at 450 °C. Reproduced from ref. [99] with permission. Copyright 2024 Elsevier.

Fan et al. [98] successfully synthesized α-Cu_2_Se thin films with uniform elemental distribution and good crystallinity, with a power factor as high as 9.23 μWcm^−1^K^−2^. Due to the many advantages of the magnetron sputtering method, the experimental group finally decided that the Se precursor was to be first prepared by thermal evaporation deposition. Then, Cu^+^ ions were injected into the prepared Se precursor by magnetron sputtering, and finally annealed at temperatures ranging from 100 °C to 240 °C intermediate to obtain α-Cu_2_Se thin films (Figure 21a). A series of tests were performed, and the HR-TEM images showed that the lamellar particles on the surface of the films became denser and denser as the temperature increased (Figure 21b,c), and it was found that the growth orientation of the α-Cu_2_Se thin films was inextricably related to the annealing temperature (Figure 21d), and finally (010) was found to be the optimal orientation. By optimizing the annealing temperature, the carrier concentration can be affected, which in turn leads to an increase in the Seebeck coefficient and a decrease in conductivity, and the maximum PF value of 9.23 μWcm^−1^K^−2^ is achieved, which is of great significance for the development of inexpensive and abundant thermoelectric films in the future. Rapaka et al. [99] also successfully synthesized Cu_2_Se thin films with excellent thermoelectric properties by changing the substrate temperature through magnetron sputtering. The process of thin film deposition is shown in Figure 21e. The change in the substrate temperature affected grain growth and electrical transport properties, etc. A series of experimental data showed that the best thermoelectric properties of Cu_2_Se thin films were obtained at a substrate temperature of 450 °C (Figure 21f,g), and the power factor of the Cu_2_Se thin films obtained by 450 °C deposition reached an amazing 16.8 μWcm^−1^K^−2^. This result will undoubtedly have a great impact on its application in the field of thermoelectricity, and magnetron sputtering is a good method to synthesize Cu_2_Se films with excellent thermoelectric properties. On the same principle, Li et al. [100] obtained Cu_2_Se-based thin films on flexible polyimide by magnetron sputtering, and through the modulation of Cu composition and growth temperature. It was finally found that the PF of the Cu/Se = 2.12 sample was higher than that of the other samples, with its maximum value of 4.12 μWcm^−1^K^−2^ at 210 °C. It is thus evident that the magnetron sputtering method is a good method and has great potential to synthesize thin films of thermoelectric materials with excellent thermoelectric properties.

Although diverse reaction conditions and synthesis strategies have been employed to optimize the thermoelectric properties of Cu_2_Se-based materials, the essence of these methods often focuses on the modulation of the crystallinity, phase composition, and the microscopic morphology of the materials. Through these fine modulations, the carrier transport properties and phonon scattering behaviors can be effectively affected, and the thermoelectric conversion efficiency of Cu_2_Se-based materials can be significantly enhanced. In short, these optimization measures aim to promote the efficient conversion of energy through fine material design, so that the Cu_2_Se-based materials can show more excellent performance in the field of thermoelectricity.

#### 3.1.3. Production of Thermoelectric Components

**Figure 22:**
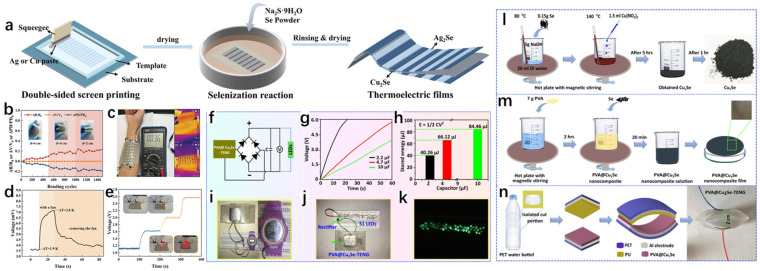
(**a**) Schematic of the synthesis of double-sided n-type Ag_2_Se and p-type Cu_2_Se films on a PI substrate; (**b**) stability of the thermoelectric device under different bending radius. The inset shows the bending photos of the thermoelectric device under different radius; (**c**) infrared images and output voltages of the thermoelectric device worn on the arm; (**d**) output voltages of the thermoelectric device of a small fan under different temperature differences; (**e**) images of the thermoelectric device lighting a diode bulb under the action of a booster. Reproduced from ref. [103] with permission. Copyright 2024 WILEY. (**f**) Circuit diagrams of charging and powering of light-emitting diode and capacitor; (**g**) capacitor charging curves; (**h**) energy stored in different capacitors; (**i**) photographs of powering of a watch; (**j**) photographic images of circuit-connected devices; (**k**) PVA@Cu_2_Se-TENG-powered light-emitting diode; (**l**) synthesis of Cu_2_Se; (**m**) formation of PVA@Cu_2_Se nanocomposite film; (**n**) schematic diagram of fabricated PVA@Cu_2_Se-TENG. Reproduced from ref. [108] with permission. Copyright 2024 Elsevier.

Cu_2_Se also has a high carrier mobility and thermoelectric figure of merit, which gives it a natural advantage in the fabrication of thermoelectric devices (TEGs). Nevertheless, it is necessary to have a high degree of flexibility in order to be used in wearable devices, in addition to its excellent thermoelectric properties. Thermoelectric devices that want to be used in wearable devices must be flexible in addition to having excellent thermoelectric properties. Studies have shown that preparing inorganic thermoelectric materials into 2D films by certain technical means is a good choice [101,102]. Zhang et al. [103] produced P-type Cu_2_Se films and n-type Ag_2_Se films by selenization using sodium sulfide hydrate and selenium powder on double-sided Ag and Cu films on flexible PI substrates by in situ synthesis (Figure 22a), which were then fabricated into a thermoelectric device to be attached to the arm. Being able to generate a voltage of 3.6 mV by utilizing the heat dissipated from the human body (Figure 22c), the device was also able to keep the LED light glowing (Figure 22e), which confirms the potential of Cu_2_Se for wearable thermoelectric device applications. In addition, Cu_2_Se can also be used in the production of generators. The conversion efficiency of the generator is mainly related to two factors: the choice of the generating material and the temperature difference between the thermoelectric parts [104]. Under this premise, to maximize the efficiency of the generator, it is necessary to have a reasonable design for its shape [105]. The friction electric nanogenerator (TENG), as an environmentally friendly device capable of converting mechanical energy into electrical energy, has attracted attention for its potential in the future [106,107]. Amini et al. [108] synthesized Cu_2_Se nanorods by the hydrothermal method (Figure 22l), and composited Cu_2_Se with PVA to fabricate PVA@Cu_2_Se-TENG in the shape of an arch bridge (Figure 22n). The composite of Cu_2_Se with PVA has certain enhancements on the dielectric constant, effective contact surface, and the crystallinity of the material, and these enhancements enable PVA@Cu_2_Se-TENG to power some electronic devices (Figure 22i,k). This study further illustrates the feasibility and necessity of Cu_2_Se for energy applications.

**Figure 23:**
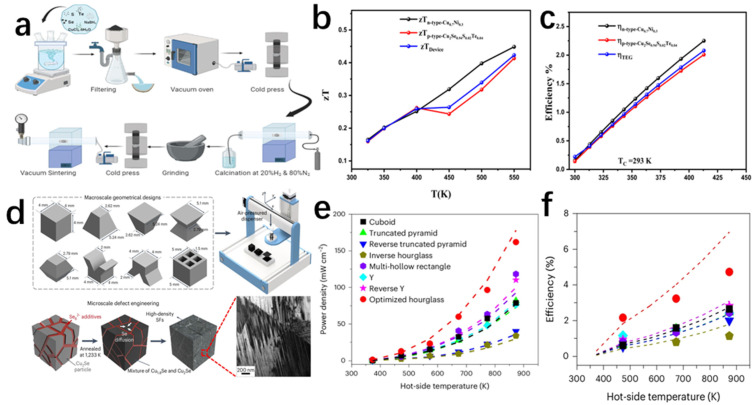
(**a**) Schematic of the synthesis of Cu_2_Se and Cu_2_Se_0.96−x_S_x_Te_0.04_ (x = 0.00, 0.01, 0.02 and 0.03) results; (**b**) images of ZT versus temperature for n-leg, p-leg, and TEG; (**c**) images of efficiency versus temperature for n-leg, p-leg, and TEG when ΔT = 120 K. The results are summarized in the following table. Reproduced from ref. [109] with permission. Copyright 2025 Royal Society of Chemistry. (**d**) 3D-printed and geometrically designed diagrams of eight different shapes of thermoelectric materials and schematic diagrams of Cu_2_Se defects; (**e**) plot of power density of 3D-printed Cu_2_Se devices with different shapes as a function of hot-side temperature in the range of 323–873 K. The power density of Cu_2_Se devices with different shapes is plotted as a function of temperature. The dashed lines represent the values simulated by the data and the dots represent the actual measured values, which fully show the consistency of the results; (**f**) plot of the efficiency of 3D-printed Cu_2_Se thermoelectric device with temperature for fixed ΔT conditions. The dashed lines represent the values simulated by the data and the dots represent the actual measured values. Reproduced from ref. [110] with permission. Copyright 2024 Springer Nature.

By doping Cu_2_Se, the same can be made better for generator fabrication. Liu et al. [109] synthesized Cu_2_Se_0.96_ by adding CuCl_2_·2H_2_O to deionized water with sulfur, tellurium, selenium powders, and the reducing agent NaBH_4_. The solution was then stirred, filtered, washed, dried, cold pressed, calcinated, and milled using the wet chemical method. Cu_2_Se_0.96−x_S_x_Te_0.04_ (x = 0.00, 0.01, 0.02, 0.03), Cu_2−y_Se_0.94_S_0.02_Te_0.04_ (y = 0.00, 0.02, 0.04), and Cu_2_Se, and the specific synthesis steps are shown in Figure 23a. The working group then constructed a thermoelectric generator (TEG) consisting of eight pairs of p-type Cu_2_Se_0.94_S_0.02_Te_0.04_ legs and n-type Cu_0.7_Ni_0.3_ legs. Due to the doping of S, the thermal conductivity and resistivity of Cu_2_Se_0.96−x_S_x_Te_0.04_ decreased, and the ZT value of the sample increased, reaching 1.54 at a temperature of 915 K when x = 0.02. The average ZT values of the p-legs, the n-legs, and the thermoelectric generator are shown in Figure 23b. In addition to this, the thermoelectric generator outputs a maximum voltage of 0.127 V when the temperature difference reaches 120 K. The maximum efficiency of the thermoelectric generator reaches 2.07% when the thermally measured temperature reaches 413 K (Figure 23c). In another work, Lee et al. [110] used advanced 3D printing techniques and 3D finite element model (FEM) simulations to determine the optimal shape of the Cu_2_Se generator. The working group first designed eight different shapes of generators (Figure 23d) and determined that the hourglass shape performed the best through a series of tests, based on which geometrical parameters of the hourglass-type generator were further optimized. This is due to the fact that dislocations are generated due to the creation of defects during the 3D printing process of liquid-phase sintering and Se evaporation, which reduces the thermal conductivity of Cu_2_Se and thus enhances the ZT value of the material, which reaches 2.0 at 950 k. The research group tested the efficiency of the generator at a fixed temperature difference and found that the hourglass shape, compared to the other shapes, has the highest efficiency and highest power (Figure 23e,f), which provides a new idea for the design of future generators—to enhance the power generation efficiency by changing the shape. However, the brittleness of the hourglass generator is not ideal, which requires further efforts from the staff to improve its performance and put it into production.

With its excellent thermoelectric properties, Cu_2_Se shows broad application prospects in the fields of flexible wearable thermoelectric devices and high-efficiency thermoelectric generators. Through composite optimization with flexible substrate materials, Cu_2_Se-based thermoelectric devices can effectively utilize the temperature difference between the human body and the environment to achieve sustained energy conversion, providing self-powered solutions for wearable electronic devices. In addition, through the precise design of the material microstructure and the combination of advanced computational simulation technology, Cu_2_Se-based thermoelectric materials are expected to break through the limitations of traditional power generation technology and become the core material for a new generation of high-efficiency thermoelectric power generation systems.

### 3.2. Energy Storage

In energy storage applications, the nanostructure design of electrode materials—such as hollow, porous, and heterojunction structures—is crucial for mitigating volume expansion and enhancing cycle stability. Template-based and ion exchange methods demonstrate unique advantages in precisely controlling nanostructures and are widely employed for preparing high-performance Cu_2_Se-based electrode materials. The following sections will highlight successful case studies of these synthesis strategies in battery applications. Cu_2_Se-based materials are a promising material for batteries due to their excellent carrier concentration and high electrical conductivity, especially in sodium-ion batteries and aqueous batteries. In addition, elemental copper and selenium are abundantly available on Earth at low cost, providing a solid foundation for their large-scale commercialization. However, Cu_2_Se-based materials are prone to structural collapse during charging and discharging, which limits their long-term cycling stability. Therefore, improving their structural stability and optimizing electrochemical performance through rational structural design (e.g., design of defects, composite or heterojunction) has become the focus of current research. In this section, the application of Cu_2_Se-based materials in energy storage and their performance optimization strategies will be systematically introduced from two aspects, namely, sodium-ion batteries and aqueous batteries.

#### 3.2.1. Sodium-Ion Battery

Transition metal selenides as battery materials have the advantages of low theoretical density, low price, more active substances, and excellent electrochemical performance [111]. On this basis, Cu_2_Se is characterized by its high conductivity and stable structure, as well as insignificant volume changes in K^+^ and Na^+^ delamination and intercalation when used as electrodes for potassium-ion batteries (PIBs) and sodium-ion batteries (SIBs), which makes Cu_2_Se excellent for use as an electrode [112].

**Figure 24:**
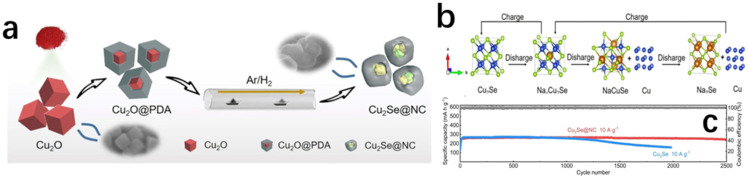
(**a**) Synthesis process of Cu_2_Se@NC; (**b**) schematic diagram of the phase transition of Cu_2_Se in charging and discharging; (**c**) comparison of the cycling performance of Cu_2_Se@NC and Cu_2_Se. Reproduced from ref. [113] with permission. Copyright 2024 Elsevier.

Wang et al. [113] used a template method, utilizing Cu_2_O as a precursor with the addition of dopamine hydrochloride, followed by calcination to obtain nitrogen-doped carbon Cu_2_Se nanomaterials (Cu_2_Se@NC) (Figure 24a). The high electrical conductivity of Cu led to an increase in the reactivity, and nitrogen doping provided more active sites for K^+^ and Na^+^. The unique structure of the composites due to the introduction of nitrogen-doped carbon resulted in a decrease in the distance between the ionic diffusion; the active material is in better contact with the electrolyte, and because of the protection of the nitrogen-doped carbon layer, the composite material is structurally stable and does not collapse easily, and has a good cycling multiplicity, which still reaches 246.8 mAh g^−1^ after 2500 cycles in a 10 A g^−1^ sodium-ion battery (Figure 24c), whereas the performance of pure Cu_2_Se material begins to show a substantial decline after 1000 cycles. The performance began to show a substantial decrease after 1000 cycles, and it is speculated that it may be due to the structural instability caused by the lack of protection of the carbon layer. The phase transition experienced by the composite material is shown in Figure 24b. The reaction equations occurring are as follows:

Cu_2_Se + xNa^+^ ↔ Na_x_Cu_2_Se (17)

Na_x_Cu_2_Se + (1 − x) Na^+^ + (1 − x) e^−^ ↔ NaCuSe + Cu (18)

NaCuSe + Na^+^+e^−^ ↔ Na_2_Se+Cu (19)

**Figure 25:**
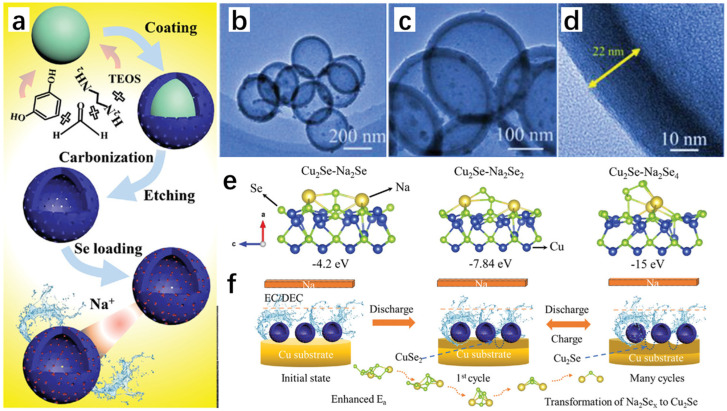
(**a**) Schematic of the preparation of fabricated Se@NHPCs; (**b**–**d**) TEM images of Se@NHPCs; (**e**) schematic of the copper collector-enhanced adsorption of polyselenide; (**f**) schematic of the copper collector-induced redox mechanism of the substitution. Reproduced from ref. [114] with permission. Copyright 2022 WILEY.

In another work, Meng et al. [114] also synthesized N-doped porous hollow spheres using the template method, and subsequently prepared Se/N-doped porous carbon spheres (Se/NHPCs) by in situ gas-phase selenization, and the flowchart of the preparation is shown in Figure 25a. In order to observe the morphology of Se/NHPCs, a series of characterization means such as SEM, TEM (Figure 25b–d) and EDS were performed on the materials. The hollow structure of the spheres with Se doping in the center can be clearly observed by these means. The Se doping is beneficial to alleviate the volume expansion during battery charging and discharging, accelerate the transport of Na^+^, and increase the attachment of more active sites. The Se/N-doped carbon sphere can be used as an in situ gas-phase selenization method. The porous hollow structure and the carbon layer can prevent the Se particles from becoming larger [115]. In addition, Se generates Se-C bonds in the process, which is favorable for charge transfer and enhances the binding energy of the carbon group with Na_2_Se. The presence of Se-C bonds has a synergistic effect on Cu_2_Se, which further enhances the Na storage function of the Se electrode, and prevents corrosion of the Cu collector to a certain extent (Figure 25f). The structure of Cu_2_Se is similar to that of Na_2_Se, and Cu_2_Se has a stronger conductivity and adsorbs Na_2_Se. The adsorption of Cu_2_Se on selenium-based compounds is related to its binding energy, and it can be seen that the binding energies of Cu_2_Se with Na_2_Se_4_, Na_2_Se_2_, and Na_2_Se are −15 eV, −7.84, and −4.2 eV, respectively (Figure 25e). As a sodium-ion anode material, the 2-Se/NHPCs electrode has an excellent reversible specific capacity and a good rate performance, with a cell capacity of 311 mAh g^−1^ at a current density of up to 5 A g^−1^, and the capacity remains at 480 mAh g^−1^ after 200 cycles at 0.5 A g^−1^.

**Figure 26:**
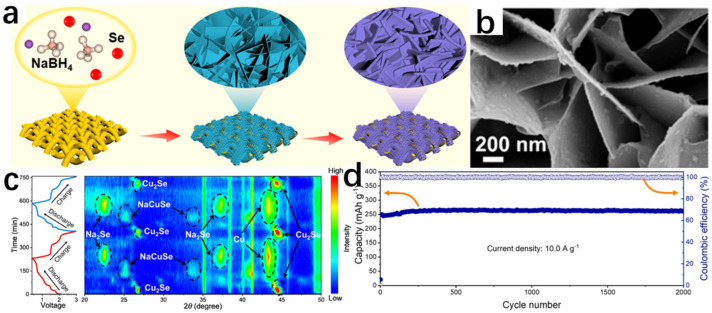
(**a**) Schematic diagram of preparation of Cu_2_Se@PPy nanosheets; (**b**) SEM images of Cu_2_Se@PPy nanosheets; (**c**) in situ XRD spectrum contour maps of two initial cycles Cu_2_Se@PPy at 0.1 A g^−1^; (**d**) rate performance of Cu_2_Se@PPy at a current density of 10.0 A g^−1^. Reproduced from ref. [116] with permission. Copyright 2022 Elsevier.

In order to alleviate the volume expansion problem of Cu_2_Se in sodium-ion batteries, a Cu_2_Se-based electrode material was prepared by Yue et al. [116]. The research group grew Cu_2_Se nanosheets on a copper mesh using NaBH_4_ as a reducing agent and selenium powder to provide Se^2−^, and subsequently prepared Cu_2_Se@PPy nanosheets by wrapping them with polypyrrole (PPy) (Figure 26a). The rougher surface of Cu_2_Se@PPy can be seen from the SEM images (Figure 26b), and the addition of PPy not only alleviated the problem of volume expansion but also improved the electrical conductivity of the composite. Combined with XRD spectrum (Figure 26c), XPS, and other test methods to analyze the charging and discharging process, the redox reactions occurred as follows:

Discharge: Cu_2_Se + Na^+^ + e^−^ → NaCuSe + Cu (20)

NaCuSe + Na^+^ + e^−^ → Na_2_Se + Cu (21)

Charge: Na_2_Se + 2Cu → Cu_2_Se + 2Na^+^ + 2e^−^ (22)

Finally, after a series of electrochemical tests, the electrochemical performance of Cu_2_Se@PPy was excellent when used as a SIB anode (Figure 26d), achieving a high specific capacity of 273.0 mAh g^−1^ (1 A g^−1^) and maintaining a specific capacity of 263.5 mAh g^−1^ (10 A g^−1^) after 2000 cycles. This simple and easy work improves the cycling stability of Cu_2_Se-based batteries to some extent and is easy to generalize.

**Figure 27:**
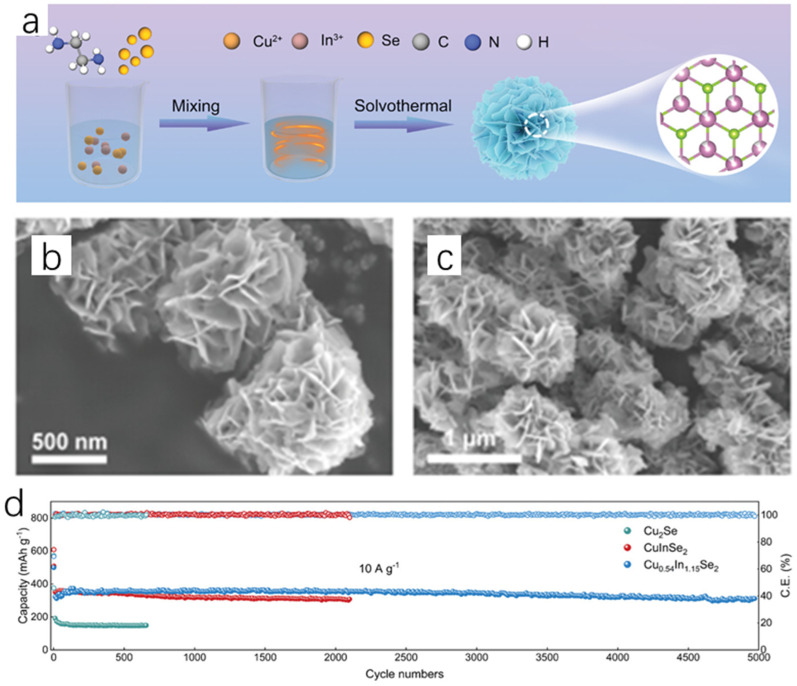
(**a**) Schematic of the synthesis of Cu_0.54_In_1.15_Se_2_ nanoflowers; (**b**,**c**) FESEM images of Cu_0.54_In_1.15_Se_2_; (**d**) cycling properties of Cu_0.54_In_1.15_Se_2_ at 10 A g^−1^ and corresponding CE. Reproduced from ref. [124] with permission. Copyright 2024 WILEY.

Defect engineering is an important method to improve the properties of SIBs [117,118,119,120]. Because defects are diverse, the addition or subtraction of atoms can be targeted to change the structure of the crystal for the purpose of performance enhancement [121]. In addition, vacancies have a beneficial effect on the stability of the phase. Sun et al. [122] synthesized CoSe_2_/FeSe_2−x_, which utilizes the excess electrons in FeS_2_ to achieve maximum adsorption and rapid diffusion of Na^+^. Since the defects tend to be disordered, this can lead to an uneven distribution of Na^+^, and the excessive buildup of some of the Na^+^ can cause an imbalance of stress in the insertion of Na^+^, which ultimately leads to structural collapse [123]. Therefore, to make the Na^+^ uniformly distributed, one can achieve that by using the method of constructing defects with an ordered distribution. Liu et al. [124] used the solvothermal method and synthesized Cu_0.54_In_1.15_Se_2_ nanoflowers in one step (Figure 27a) and based on the field emission scanning electron microscopy (FESEM) images, it can be determined that the nanoflowers are composed of nanosheets with a diameter of about 1 nm (Figure 27b,c). EDX testing of the material showed that the three elements, Cu, ln, and Se, are uniformly distributed in the material, which may have a positive effect on the uniform distribution of Na^+^ and make the structure of the material less likely to collapse. The Cu vacancies and ordered defect pairs (lnCu) contained in Cu_0.54_In_1.15_Se_2_ affect the adsorption of Na^+^ by acting on the Se atoms in the material and thus, the conductivity of the material increases. Under the action of vacancies, Na^+^ moves uniformly in all directions, and the accumulation of Na^+^ is avoided, and the uniform distribution of Na^+^ is conducive to the stabilization of the structure. After 5000 cycles, Cu_0.54_In_1.15_Se_2_ still maintains a reversible capacity of 311.7 mAh g^−1^ and a high retention rate of 89.2% as a cathode material for sodium-ion batteries at a current density of 10 A g^−1^ (Figure 27d), which is much higher than that of Cu_2_Se and CulnSe_2_. While the CulnSe_2_ electrode is not as good as Cu_2_Se and CulnSe_2_ due to the absence of organized Cu vacancies, which led to the occurrence of Na^+^ buildup and the shedding of the electrode volume expansion during the cell operation. In the end, the CulnSe_2_ electrode could not be used after only 2000 cycles. This work strongly demonstrates that the construction of ordered vacancies is conducive to the improvement of the electrochemical performance of the anode and the stability of the electrode structure in sodium-ion batteries.

It has been shown that the electrochemical properties of materials can be enhanced by designing special heterojunction structures. Under the effect of heterojunction, two semiconductors with different bandgaps will form an interface with an internal electric field, and the ion diffusion and charge transfer are significantly enhanced, and the reaction kinetics are strengthened [125,126]. At the same time, the electrical conductivity of the material is enhanced, the volume expansion problem is alleviated, and the lifetime and cycling stability of the electrode are optimized. In addition, the electrochemical properties of the materials can also be substantially improved by designing the microforms of the materials.

**Figure 28:**
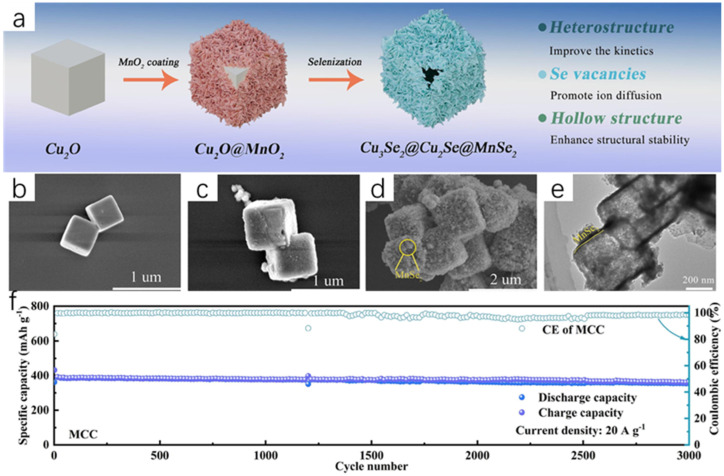
(**a**) Schematic of MCC nanotube preparation; (**b**) FESEM image of precursor Cu_2_O; (**c**) FESEM image of MnO_2_@Cu_2_O; (**d**) FESEM image of MCC; (**e**) TEM image of MCC; and (**f**) long cycle stability of MCC at high current density of 20 A g^−1^. Reproduced from ref. [127] with permission. Copyright 2025 Elsevier.

Yang et al. [127] designed a MnSe_2_@Cu_3_Se_2_/Cu_2_Se (MCC) hollow nanotube by using co-precipitation method and selenization. The composite material has a unique heterogeneous structure and selenium vacancies; the heterogeneous structure improves the reaction kinetics; the Se vacancies accelerate the diffusion of ions; and the hollow structure prevents the material from collapsing, which is beneficial for the stability of the material. The preparation process is shown in Figure 28a. Firstly, Cu_2_O nanocubes were prepared by the co-precipitation method (Figure 28b), then Cu_2_O was wrapped with MnO_2_ nanosheets, and finally the core–shell structure Cu_2_O@MnO_2_ (Figure 28c) was selenized to form MnSe_2_@Cu_3_Se_2_/Cu_2_Se (MCC) nanocubes with hollow structure (Figure 28d). Due to the superiority of the structure, the MCC still has a high capacity of 358 m Ah g^−1^ and a retention rate of 98% after 3000 charges and discharges at a high current density of 20 A g^−1^ (Figure 28f), which fully demonstrates that the design of the heterogeneous structure and the unique hollow structure is of great significance for the electrochemical performance and cycling stability of the electrode materials. The reaction and phase transition processes experienced by MCC in sodium-ion batteries can be inferred after XRD spectrum, XPS and other related tests:

Discharge process equation:

3Cu_3_Se_2_ + 2e^−^ + 2Na^+^ → 5Cu_1.8_Se + Na_2_Se (23)

10Cu_1.8_Se + 2e^−^ + 2Na^+^ → 9Cu_2_Se + Na_2_Se (24)

Cu_2_Se + 2e^−^ + 2Na^+^ → 2Cu + Na_2_Se (25)

MnSe_2_ + e^−^ + Na + → NaMnSe_2_ (26)

NaMnSe_2_ + 2e^−^ + Na^+^ → Mn + Na_2_Se (27)

Charge process equation:

2Cu + Na_2_Se → Cu_2_Se + 2e^−^ + 2Na^+^ (28)

Mn + Na_2_Se → NaMnSe_2_ + 2e^−^ + 2Na^+^ (29)

Moreover, the full cell assembled by the researchers with MCC as the anode and Na_3_V_2_(PO_4_)_3_@C as the cathode also has excellent multiplicative performance, with a high capacity of 161 mAh g^−1^ after 1000 cycles at a current density of 10 A g^−1^. The full cell is also capable of fast charging and discharging, requiring only 60 s to complete the charging process, and was subsequently tested to light up a string of LED bulbs, demonstrating its potential application value.

**Figure 29:**
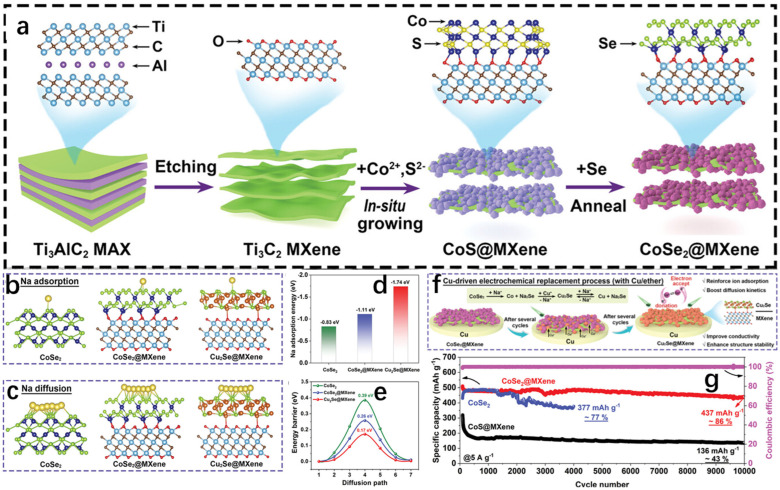
(**a**) Schematic diagram of the synthesis of CoSe_2_@MXene; (**b**–**e**) diagrams of CoSe_2_, CoSe_2_@MXene, and Cu_2_Se@MXene in relation to Na+, (**b**) adsorption diagram of Na atoms; (**c**) side view of the diffusion pathway of Na+; (**d**) co-phase sodium adsorption energies; (**e**) co-phase diffusion energy barriers; (**f**) Na+ storage in Cu_2_Se@MXene in Cu-driven electrochemical substitution process; (**g**) performance diagram of CoSe_2_@MXene, pure CoSe_2_, and CoS@MXene electrodes under 10,000 cycles at a current density of 5 A g^−1^. Reproduced from ref. [128] with permission. Copyright 2025 WILEY.

Not coincidentally, Lu et al. [128] prepared sandwich-type CoSe_2_@MXene composites using an etching process and a selenization process (Figure 29a), and based on this, they designed Cu_2_Se@MXene heterostructures using electrochemical reactions. Mxenes are 2D materials with highly conductive and stable layered structures whose main components are transition metal carbon nitrides, and they have been widely used in the electrochemical fields [129,130,131]. Mxenes are often prepared by etching, in which a large number of groups are introduced, so they tend to have excellent charge transfer capabilities [132,133]. Cu_2_Se has a natural advantage in Na^+^ storage due to its high ion mobility [134]. CoSe_2_@MXene has a high capacity and excellent multiplicity performance when used as an anode for sodium-ion batteries. This is demonstrated by a capacity of 694 m Ah g^−1^ at a current density of 0.1 A g^−1^, and a capacity of 437 mAh g^−1^; 86% of the original capacity was maintained after 10 000 cycles at a current density of 5 A g^−1^ (Figure 29g). Such an excellent performance may be the result of a combination of the following factors: the unique two-dimensional structure of Mxenes avoids volume expansion and improves the interfacial contact area; the CoSe_2_@MXene heterojunction is rich in active sites, which promotes Na^+^ adsorption and the subsequent generation of new active sites; the presence of Cu_2_Se, which further enhances the diffusion and adsorption of Na^+^. This can also be verified by Figure 29d,e. Figure 29d shows that the adsorption energy of Cu_2_Se@MXene is −1.74 ev, which is much lower than that of CoSe_2_@MXene and CoSe_2_. This is due to the large number of active check points of Cu_2_Se on the heterojunction surface, which enhances the adsorption of Na. In Figure 29e, it is found that the diffusion barrier energy of Cu_2_Se@MXene is also much lower than that of CoSe_2_@MXene and CoSe_2_, which may be due to the joint action of Mxene and Cu_2_Se. Figure 29f reveals the storage process of Na + and the reaction of Cu during the displacement reaction. This work once again demonstrates that the construction of specially structured heterojunctions plays an indispensable role in optimizing the electrochemical properties of materials. In addition, on this path of constructing special heterojunctions, Tian et al. [135] synthesized porous octahedral MoSe_2_-Cu_1.82_Se@GA (graphene aerogel). The special octahedral structure facilitates the increase in electrical conductivity and mitigates the volume change in the material during charging and discharging. The porous structure facilitates the transport of Na^+^, and the presence of heterojunctions drastically reduces the activation energy of charge transfer, which promotes charge diffusion as well as the storage of Na^+^. The results showed that a specific capacity of 444.8 mAh g^−1^ was achieved at a current density of 1 A g^−1^ when MoSe_2_-Cu_1.82_Se@GA was used as the anode of the SIBs. In the future, it will be promising to extend the work to construct bimetallic selenides/GA as anodes for SIBs with excellent electrochemical properties.

It is not difficult to see that Cu_2_Se-based materials exhibit excellent electrochemical performance in sodium-ion batteries (SIBs) mainly due to their high electrical conductivity, abundant copper vacancies, and good structural stability. By introducing nitrogen-doped carbon layers, constructing ordered defects or designing heterojunction structures, the specific capacity and cycling stability of Cu_2_Se-based materials are significantly enhanced. In addition, by constructing ordered vacancies and heterojunctions, the Cu_2_Se-based materials were further optimized for the application in sodium-ion batteries, exhibiting higher specific capacity and longer cycle life. Future research could focus on further optimizing the microstructure of Cu_2_Se-based materials, such as developing more complex heterojunctions or porous structures, to enhance their electrochemical performance. Meanwhile, the development of high-performance composites in combination with novel conductive materials (e.g., MXene or graphene) will promote the large-scale application of Cu_2_Se-based materials in sodium-ion batteries.

#### 3.2.2. Water-Based Battery

With the in-depth popularization of the concept of carbon-free energy saving and environmental protection, the demand for energy storage devices with high safety, low cost, and high energy density has reached an unprecedented high level [136,137]. Water-ion batteries have many advantages in this regard, such as low pollution, low cost, high safety, and stable sources, which have received widespread attention [138,139]. Cu_2_Se is rich in content, has low cost, and is low in pollution. In addition, it has a narrow bandgap, which is favorable for the carrier mobility; Cu^2+^ has a high redox property, which always plays an important role in the energy storage process, and these advantages make Cu_2_Se an important material for aqueous batteries [140].

**Figure 30:**
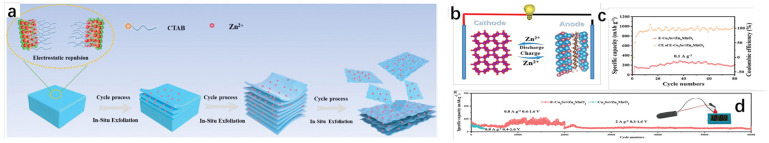
(**a**) Schematic diagram of stripping of E-Cu_2_Se; (**b**) schematic diagram of charging and discharging of Zn_x_MnO_2_||E-Cu_2_Se battery; (**c**) cycling performance diagram of Zn_x_MnO_2_||E-Cu_2_Se battery; (**d**) cycling performance diagram of Zn_x_MnO_2_||E-Cu_2_Se battery and photographs of two Zn_x_MnO_2_||E-Cu_2_Se batteries in series positive use. Reproduced from ref. [141] with permission. Copyright 2024 WILEY.

Bai et al. [141] obtained Cu_2_Se nanosheets (E-Cu_2_Se) through an in situ exfoliation mechanism in an electrochemical reaction (Figure 30a) and used it as the anode, and Zn_x_MnO_2_ is used as the cathode in an aqueous zinc-ion battery (Figure 30b). Due to the stability of the Cu_2_Se structure and its excellent performance, problems such as Zn dendrite generation and corrosion of the metal anode are greatly reduced and showed good cycle multiplication with high specific capacity (Figure 30c,d), with the performance still exhibiting 40 mAh g^−1^ after 60,000 cycles. This work once again strongly confirms the potential of Cu_2_Se application in batteries. In another work, Guo et al. [142] synthesized an aqueous zinc-selenium battery with a porous structure, and the main material of the battery is N-P asymmetrically coordinated copper monoatom (CuN_3_P_1_@C). Due to the high loading of copper monoatom, the asymmetry of the coordination environment, and the porous structure of the material, the reaction barrier of the redox reaction is greatly reduced, and the electrochemical performance of the CuN_3_P_1_@C battery is excellent. The electrochemical performance of the CuN_3_P_1_@C cell is excellent. The specific capacity of Se/CuN3P1@C cathode material reaches a high level of 756 mAh g^−1^ at a current density of 0.2 A g^−1^ and remains 181 mAh g^−1^ at a current density of 5.0 A g^−1^, with a retention rate of 82.3%.

**Figure 31:**
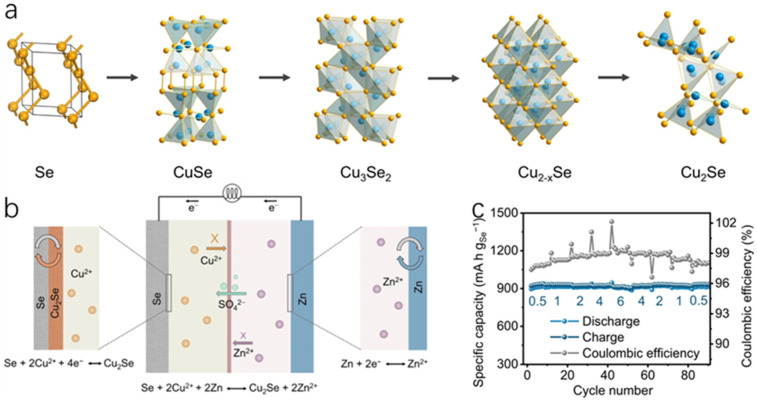
(**a**) Schematic diagram of the phase transition of the Se structure during discharge; (**b**) schematic diagram of the aqueous Zn||Se@C-48 cell; (**c**) fast charging rate performance plots of the aqueous Zn||Se@C-48 cell: charging at 6 A g^−1^ and discharging at different currents. Reproduced from ref. [143] with permission. Copyright 2022 Springer Nature.

In order to improve the cycling performance of selenium-based materials at the cathode, Dai et al. [143] synthesized a selenium@carbon (Se@C) cathode material. When the electrolyte contains Cu^2+^, the solution will undergo a series of redox reactions when it comes into contact with Cu or Zn (Figure 31b). Se is converted to Cu_2_Se by reacting with Cu^2+^ at the cathode, and Zn is oxidized by losing electrons at the anode as Zn^2+^. Se undergoes a series of phase transitions at the cathode as shown in Figure 31a (Se ↔ CuSe ↔ Cu_3_Se_2_ ↔ Cu_2−x_Se ↔ Cu_2_Se). In this work, in order to better illustrate the electrochemical performance of the Se@C electrode, the researchers fabricated a homemade aqueous Zn||Se@C-48 full cell with an electrolyte containing 0.5 M ZnSO_4_ and 0.5 M CuSO_4_. The full cell has a fast charging and discharging capability and is charged at a current density of 6 A g^−1^ with a specific capacity of about 900 mAh g^−1^. After 400 cycles, the Coulombic efficiency was still greater than 98% (Figure 31c). These data strongly demonstrate the great potential of the Zn||Se@C-48 full cell for fast charging and discharging when put into practical applications.

**Figure 32:**
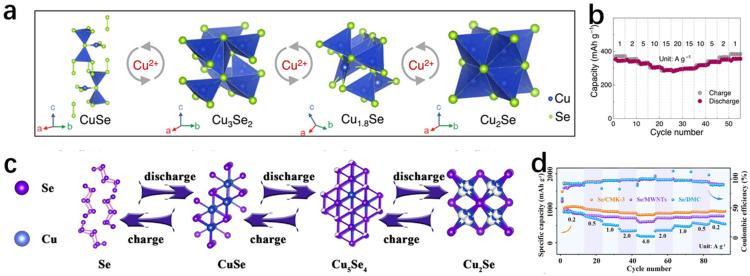
(**a**) Schematic of the phase transition of CuSe during charging and discharging; (**b**) variation in the rate capability of CuSe with current rate (from 1 to 20 A g^−1^). Reproduced from ref. [138] with permission. Copyright 2022 WILEY. (**c**) Schematic of the phase transition in Cu-Se cells; (**d**) rate capability of Se/DMC, Se/MWNTs and Se/CMK-3. Reproduced from ref. [144] with permission. Copyright 2023 Elsevier.

CuSe is rich in Cu vacancies, which can be converted to Cu_2_Se through redox reactions, and it is an ideal material for batteries due to its excellent conductivity and the large number of active sites attached to it, which allows it to undergo rapid charge transfer. Wang et al. [138] prepared CuSe nanosheets by simple hydrothermal and ion exchange methods and underwent a series of redox reactions during its charging and discharging processes. Reactions, and the reversible phase transition from CuSe to Cu_3_Se_2_ to Cu_1.8_Se_2_ to Cu_2_Se, was revealed by XRD spectrum (Figure 32a). Meanwhile, due to the excellent ability of Cu^2+^ ions in redox reactions, the cell has excellent rate capacity (Figure 32b) and cycling stability, maintaining a high performance of 285 mA h g^−1^ after 20 A g^−1^, and this performance only decreases by 10% after 30,000 cycles. However, Se-based batteries have the problem of volume expansion when used as the cathode of the battery, leading to poor cycling performance and low electrochemical performance. Lin et al. [144] developed a novel Cu-Se aqueous battery to solve this problem, which utilizes selenium encapsulated in ordered mesoporous carbon (Se/CMK-3) as the cathode, which undergoes a series of redox reactions during charging and discharging (Figure 32c):

Cathode:

Se + Cu^2+^ + 2e^−^ → CuSe (30)

CuSe + 0.25Cu^2+^ + 0.5e^−^ → 0.25Cu_5_Se_4_ (31)

0.25Cu_5_Se_4_ + 0.75Cu^2+^ + 1.5e^−^ → Cu_2_Se (32)

Anode:

2Cu → 2Cu^2+^ + 4e^−^ (33)

Overall reaction:

Se + 2Cu → Cu_2_Se (34)

In order to better highlight the superiority of Se/CMK-3, the experimental group also prepared selenium/multi-walled carbon nanotubes (Se/MWNTs) and selenium/disordered mesoporous carbon (Se/DMC) composites as controls. Due to the special structure of Se/CMK-3 suppressing the volume change during charging and discharging, and its abundant active sites, the Cu-Se cell has excellent electrochemical performance (Figure 32d). The novel Cu-Se aqueous cell has a high capacity of 1036 mAh g^−1^ at 0.2 A g^−1^ and excellent cycling stability (93.7%) after 400 cycles at 4.0 A g^−1^. These two works fully demonstrate the potential of CuSe-based compounds in the development of aqueous batteries with excellent electrical capacity and cycling stability. There are many similar works, such as Xu et al. [145] who developed a Cu-Se aqueous battery, in which the specific capacity of Se at the cathode is enhanced due to the change of Cu^2+^ to Cu^+^ during charging and discharging. The cell has a reversible specific capacity of 1045.4 mAh g^−1^ at a current density of 0.1 A g^−1^ and only loses 10.3% of its capacity at 2 A g^−1^ for 1500 cycles.

In water-based battery, Cu_2_Se-based materials exhibit excellent electrochemical performance due to their high electrical conductivity and good redox properties. By designing the porous structure and introducing heterojunctions, Cu_2_Se-based materials exhibit high specific capacity and long cycle life in aqueous zinc-ion batteries. By introducing a copper monoatomic catalyst and a porous structure, the reaction kinetics of Cu_2_Se-based materials in aqueous batteries were significantly enhanced, further improving the energy density and cycle life of the batteries. Future studies can further enhance the performance of Cu_2_Se-based materials in aqueous batteries, such as optimizing the electrochemical properties of the materials by constructing more complex heterojunctions or porous structures

## 4. Summary and Prospects

Cu_2_Se-based materials show great potential for application in the field of energy conversion and storage due to their excellent thermoelectric properties, high electrical conductivity and abundant resources. Energy conversion and storage are two key aspects of energy systems, and they are complementary to each other. Cu_2_Se-based materials convert waste heat into electricity through the Seebeck effect in thermoelectric conversion, and store electricity through electrochemical reactions in energy storage. This synergistic relationship not only improves the efficiency of energy utilization, but also provides the possibility of developing multifunctional devices (e.g., integrated thermoelectric-energy storage devices), which further promotes the development of green energy technologies. This manuscript reviews various synthesis methods of Cu_2_Se-based materials (solid-phase synthesis, hydrothermal method, ion exchange method, etc.) and their applications in energy conversion and energy storage. Although Cu_2_Se-based materials have made some achievements in energy conversion and storage, there are still some shortcomings. Although Cu_2_Se is an excellent lead-free alternative material, elemental selenium and some of its compounds exhibit certain toxicity. This necessitates strict adherence to safety protocols during research, manufacturing, and the final disposal of devices. Throughout experiments, appropriate personal protective equipment (PPE) must be worn, and synthesis operations should be conducted in well-ventilated fume hoods, and proper waste management procedures must be implemented. It is believed that Cu_2_Se-based materials will have better development through the following measures:

1. Material property optimization: Further enhancing the thermoelectric and electrochemical properties of Cu_2_Se-based materials through the development of more complex heterojunctions, porous structures or ordered defects.
2. Novel composite material development: Develop multifunctional energy devices, such as integrated thermoelectricity-energy storage devices, with composite Cu_2_Se with high-performance materials, such as graphene and MXene.
3. Green synthesis process: Explore low-cost, high-efficiency, and environmentally friendly synthesis methods, such as 3D printing or cold sintering technology, to promote the large-scale commercialization of Cu_2_Se-based materials.
4. Multifunctional integrated appliances: Combine with advanced computational simulation techniques, and design new device structures to further enhance the application potential of Cu_2_Se-based materials in the fields of wearable devices and industrial waste heat recovery.

With these technological breakthroughs, Cu_2_Se-based materials are expected to realize a wider range of applications in the field of energy conversion and storage, providing important support for the development of green energy technology.

## Figures and Tables

**Table 1:** Summary of advantages and disadvantages of template-directed, chemical reduction, solid-phase synthesis, hydrothermal/solvothermal method, and ion exchange methods.

Synthesis Methods	Advantages	Disadvantages
Template-guided method	Precise control of the size and shape of the product, so as to achieve precise control of the product; the synthesized new material can well retain the size and shape of the raw material.	Difficult to select templates, cumbersome, and difficult to operate.
Chemical reduction	Simple to operate; no need for expensive equipment and tedious steps; easy to control.	Introduces new impurities that are difficult to remove and pollutes the environment.
Solid-phase method	High yield of prepared powder; basically no agglomeration phenomenon; good filling; low production cost; simple and easy to master the technology.	Low reaction efficiency; high energy consumption; large powder particles; often mixed with impurities in the preparation process.
Hydrothermal/solvent-thermal	Sintering is not required during the reaction process, which can avoid excessive growth of grains and the mixing of impurities; the synthesized particles are well-dispersed, with high purity; the microscopic morphology of the particles is good and controllable, which does not require high cost.	The growth process of the sample cannot be visualized in a closed environment; requires certain equipment (corrosion-, high temperature-, and pressure-resistant); requires certain technology; requires strict control of temperature and pressure.
Ion exchange method	The morphology of precursor can be well preserved; the process is simple, easy to operate, causing less pollution.	Difficult to remove impurities during the synthesis process; long synthesis period, etc.

## Data Availability

Not applicable.
